# Angiogenesis-Informed Preoperative CT Radiogenomics Predicts Overall Survival in Clear Cell Renal Cell Carcinoma: Development and External Validation

**DOI:** 10.3390/cancers18050768

**Published:** 2026-02-27

**Authors:** Yanghuang Zheng, Yuelin Du, Zhongwei Ma, Yao Luo, Jianzhong Lu, Panfeng Shang

**Affiliations:** 1 Department of Urology, Gansu Province Clinical Research Center for Urinary System Disease, The Second Hospital & Clinical Medical School, Lanzhou University, No. 82 Cuiyingmen, Lanzhou 730030, China; zhengyh2024@lzu.edu.cn (Y.Z.); duyl21@lzu.edu.cn (Y.D.); mazhw2023@lzu.edu.cn (Z.M.); 120220902071@lzu.edu.cn (Y.L.); 2 Institute of Urology, Gansu Province Clinical Research Center for Urinary System Disease, The Second Hospital & Clinical Medical School, Lanzhou University, No. 82 Cuiyingmen, Lanzhou 730030, China; lujzh@lzu.edu.cn

**Keywords:** clear cell renal cell carcinoma, machine learning, prediction, prognosis, radiogenomics

## Abstract

Background/Objectives: We aimed to identify angiogenesis-related prognostic biomarkers and develop a radiogenomics model to predict overall survival (OS) in clear cell renal cell carcinoma (ccRCC), supporting risk stratification and potential therapeutic target discovery. Methods: Bulk transcriptomes from The Cancer Genome Atlas Kidney Renal Clear Cell Carcinoma cohort (TCGA-KIRC), seven Gene Expression Omnibus (GEO) microarrays, and a single-cell RNA sequencing (scRNA-seq) dataset were integrated to identify angiogenesis-related prognostic genes. Preoperative contrast-enhanced computed tomography (CT) images from The Cancer Imaging Archive Kidney Renal Clear Cell Carcinoma collection (TCIA-KIRC) were used for radiomics feature extraction, and a radiogenomics signature was constructed by linking radiomic features with transcriptomic risk patterns. Nine machine learning models were trained to predict OS; the best model was further evaluated in an independent external retrospective cohort. PDLIM1 (PDZ and LIM domain protein 1) was validated at the protein level, and conditioned medium from stable ccRCC cell lines was applied to human umbilical vein endothelial cells (HUVECs) for Matrigel tube formation assays. Results: Five angiogenesis-related hub genes (PDLIM1, EMCN, ARPC1B, PLAT, and TIMP1) were identified. The extreme gradient boosting (XGBoost)-based radiogenomics model showed the best performance, with time-dependent concordance index (C-index) values of 0.880, 0.816, and 0.789 at 1, 3, and 5 years in the training set and 0.864, 0.758, and 0.736 in the internal validation set, respectively. In the external validation cohort, C-index values were 0.800, 0.726, and 0.703 at 1, 3, and 5 years. PDLIM1 protein was upregulated in ccRCC versus normal tissues. Functionally, PDLIM1 overexpression suppressed, whereas PDLIM1 knockdown promoted tube formation. Conclusions: This study developed and validated an angiogenesis-related radiogenomics model that accurately predicts OS in ccRCC patients and provides potential therapeutic targets for anti-angiogenic therapy.

## Simple Summary

Clear cell renal cell carcinoma (ccRCC) is a highly vascular tumor, and angiogenesis plays a central role in its progression and response to therapy. However, reliable tools that combine imaging and molecular information to predict patient survival and reveal actionable targets are still needed. In this study, we integrated bulk and single-cell transcriptomic datasets to identify angiogenesis-related genes associated with overall survival, and we linked these risk patterns to radiomics features extracted from preoperative contrast-enhanced CT scans. Using multiple machine learning approaches, we developed a radiogenomics model and found that an XGBoost-based prediction model achieved the best and consistent performance across internal and external validation cohorts. We further confirmed elevated PDLIM1 protein expression in ccRCC tissues and demonstrated that PDLIM1 can suppress endothelial tube formation in functional assays. Overall, our angiogenesis-related radiogenomics model enables effective risk stratification for ccRCC patients and highlights potential therapeutic targets for anti-angiogenic treatment.

## 1. Introduction

Renal cell carcinoma is a malignant tumor that originates from the epithelial cells of the renal tubules, with clear cell renal cell carcinoma (ccRCC) constituting approximately 70% of renal cell carcinoma (RCC) cases [1]. For patients diagnosed with localized renal cell carcinoma (stages I–III), radical nephrectomy serves as the primary surgical treatment [2]. Nevertheless, a substantial proportion of patients experience postoperative recurrence/metastasis or present with advanced disease, resulting in poor long-term outcomes [3,4].

The von Hippel–Lindau (VHL) tumor suppressor is frequently altered in ccRCC. Loss of VHL function leads to aberrant stabilization of hypoxia-inducible factors (HIF-1α/HIF-2α), which promotes angiogenesis and contributes to the highly vascular phenotype of ccRCC [5,6]. Although vascular endothelial growth factor (VEGF) pathway inhibitors and immune checkpoint blockade have improved survival in some patients, response rates remain limited and tumor heterogeneity contributes to therapeutic resistance and disease progression [7,8,9].

Radiogenomics is an emerging discipline that aims to correlate imaging features with gene expression patterns, gene mutations, and other genomically relevant characteristics, thereby enhancing the understanding of tumor biology and capturing inherent tumor heterogeneity [10]. In the era of precision medicine, the high cost of genomic sequencing poses accessibility challenges for many patients, while the interpretation of tumor biology based on imaging phenomics has its limitations [11]. Radiogenomic analysis can reflect biological processes at genetic and molecular levels through biomedical images, and parameters from advanced image processing can reveal underlying phenotypic and genotypic tissue characteristics [12]. These features enable deeper insights into tumor biology while effectively capturing intra-tumoral heterogeneity [13,14]. Consequently, by integrating imaging-derived features with genomic assays, radiogenomic biomarkers can facilitate diagnosis, prognostic assessment, and therapy stratification while mitigating the cost and accessibility barriers of widespread molecular testing.

Currently, imaging–molecular biomarkers obtained through radiogenomic analysis are utilized in developing diagnostic and survival prediction models for tumors, primarily focusing on gliomas, lung cancer, and breast cancer [12]. Caruso et al. developed a predictive model for high-risk colon cancer using radiogenomic data, achieving an area under the receiver operating characteristic curve (AUC) of 0.84 (95% confidence interval [CI]: 0.68–0.99), which outperformed single radiomics models [15]. Similarly, Li et al. demonstrated that their developed radiogenomic predictive model exhibited strong performance in predicting the response of neck and head squamous cell carcinoma patients to induction chemotherapy, with an AUC of 0.88 (95% CI: 0.75–0.96) [16]. While multi-data integration analysis has gained traction, there remains limited research on imaging–molecular biomarker predictive models targeting angiogenesis in ccRCC.

Despite the availability of multiple prognostic models for ccRCC, their predictive performance and clinical generalizability still warrant improvement [3]. When used in isolation, clinical pathological variables or radiomics features provide limited biological interpretability and may be influenced by differences in imaging protocols and institutions [17]; conversely, genomics-only signatures depend on tissue acquisition and sequencing resources and may be biased by intratumoral heterogeneity [18]. Angiogenesis-related biomarkers represent a key tumor state in ccRCC that cannot be fully captured by morphology-based stratification or imaging phenotypes alone. Therefore, an angiogenesis-focused radiogenomic framework linking computed tomography (CT)-derived radiomic features with angiogenesis-associated molecular signatures may provide incremental prognostic value by enhancing interpretability and enabling noninvasive, whole-tumor characterization.

In this study, we integrated bulk RNA sequencing (RNA-seq) data from The Cancer Genome Atlas Kidney Renal Clear Cell Carcinoma cohort (TCGA-KIRC), multiple microarray datasets from The Gene Expression Omnibus (GEO), and single-cell RNA sequencing (scRNA-seq) data to identify angiogenesis-related prognostic biomarkers in ccRCC. We then extracted radiomics features from preoperative contrast-enhanced CT images from The Cancer Imaging Archive Kidney Renal Clear Cell Carcinoma collection (TCIA-KIRC) and developed machine learning-based radiogenomics models for overall survival (OS) prediction, followed by external validation in an independent retrospective cohort. Finally, we performed protein-level validation of PDLIM1 (PDZ and LIM domain protein 1) and conducted functional assays to evaluate its potential involvement in tumor-driven angiogenic activity. Based on the premise that CT radiomic phenotypes can serve as noninvasive surrogates of angiogenesis-related molecular programs, we further assessed whether integrating these imaging features with angiogenesis-associated biomarkers improves OS risk stratification in ccRCC.

## 2. Materials and Methods

**Figure 1:**
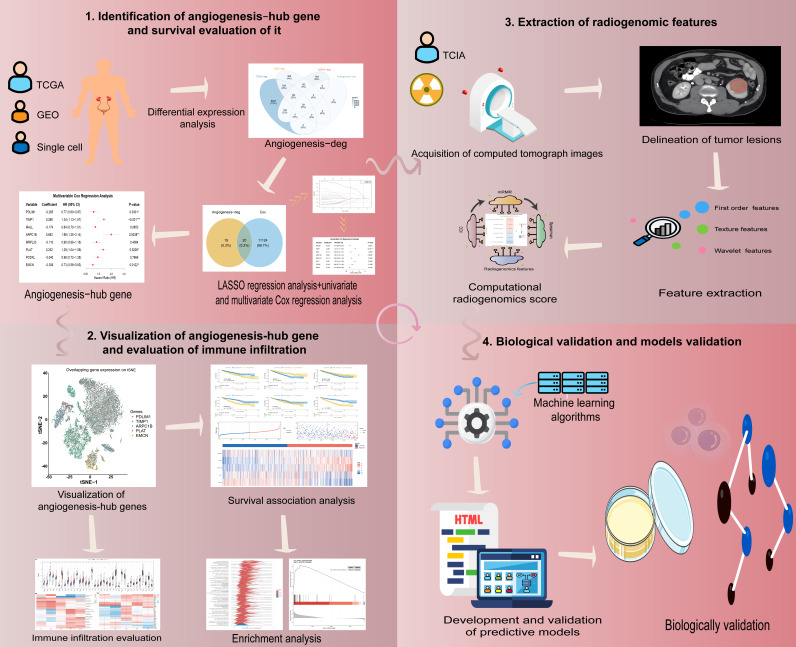
The overall flowchart of this study.

This study was conducted in four major phases. First, bulk transcriptomic profiles from the TCGA-KIRC cohort and multiple GEO microarray datasets, together with scRNA-seq data, were integrated to identify angiogenesis-related prognostic genes and derive a prognosis-associated angiogenesis signature in ccRCC. Second, after establishing this signature, we performed functional annotation and pathway analyses (Kyoto Encyclopedia of Genes and Genomes (KEGG) and Gene Ontology (GO) enrichment and network visualization) to characterize the underlying biological processes. Third, preoperative contrast-enhanced CT images from TCIA-KIRC were used for radiomic feature extraction, and a radiogenomics score was constructed by linking radiomic phenotypes to the angiogenesis-related risk signature. Finally, machine learning-based prognostic models incorporating the radiogenomics score and clinicopathological variables were developed in the training cohort, internally validated, and further tested in an independent external retrospective cohort; the key gene PDLIM1 was validated by Western blotting (WB), and its functional relevance to tumor-driven angiogenesis was assessed using conditioned medium-based human umbilical vein endothelial cell (HUVEC) tube formation assays. An overview of the study design is shown in Figure 1.

### 2.1. Data Collection

Seven transcriptome microarray datasets of ccRCC were obtained from the GEO database, including GSE68417, GSE76351, GSE46699, GSE5300, GSE73757, GSE36895, and GSE14641, along with one scRNA-seq (GSE242299). In addition, bulk transcriptome data for ccRCC, including TPM expression matrices, raw count data, somatic mutation data, survival information, and clinical baseline characteristics, were obtained from the TCGA-KIRC cohort via the TCGA and UCSC Xena portals. Gene annotation files provided by UCSC Xena were used to map Ensemble IDs to gene symbols. Cases without survival data and those with survival time less than 30 days were excluded. Clinical baseline variables included age, sex, AJCC stage, Fuhrman grade, tumor laterality, race, and OS data.

Preoperative contrast-enhanced CT images of ccRCC were obtained from the TCIA-KIRC collection and matched with TCGA-KIRC patients for radiomics feature extraction and radiogenomics analysis [19]. Furthermore, a retrospective cohort of ccRCC patients from the Second Hospital of Lanzhou University (January 2017 to December 2024) was included as an external validation cohort. The inclusion criteria were as follows: (1) pathologically confirmed ccRCC; (2) availability of clinical baseline characteristics including age, sex, stage (American Joint Committee on Cancer (AJCC)), Fuhrman grade, tumor laterality, race, and OS; and (3) preoperative abdominal contrast-enhanced CT performed within two weeks before surgery. Exclusion criteria were: (1) poor image quality; (2) inability to delineate the tumor lesion; (3) receipt of any antitumor therapy prior to imaging; and (4) without follow-up survival data. Follow-up was scheduled every 3–6 months during the first 2 years after surgery and annually thereafter, until December 2024 or the last follow-up, with clinical assessments including physical examinations, imaging studies (CT or magnetic resonance imaging [MRI]), and laboratory tests. The follow-up duration ranged from 6 months to 5 years, and patients at higher risk for recurrence or metastasis were monitored more frequently. The detailed recruitment process is shown in Supplementary Figure S1.

### 2.2. Transcriptome Data Analysis

Gene annotation information for the GEO microarray data was obtained using the R package tinyarray (v1.0.0), and samples were classified into tumor and normal tissue groups. Batch effects across the seven microarray datasets were removed using the R package sva (v3.42.0), and principal component analysis (PCA) was applied to visualize sample clustering before and after correction. Differentially expressed genes (DEGs) between tumor and normal tissues were identified using the limma algorithm [20].

For the TCGA-KIRC bulk RNA-seq data, differential expression analysis was carried out using three algorithms: limma, DESeq2, and edgeR. Only overlapping DEGs identified by all three methods were retained [21]. DEGs were defined as those with |log2 fold change| > 1 and adjusted *p* value < 0.05.

### 2.3. Single-Cell RNA-Seq Analysis

The scRNA-seq count matrix of GSE242299 was processed using Seurat (v4.3.0), followed by quality control. Genes expressed in at least three cells and cells with at least 300 detected genes were retained. Cells with a mitochondrial RNA percentage greater than 20% were excluded. Data were normalized using the LogNormalize method.

The top 2000 highly variable genes were identified using the VST method. Data were scaled, and batch effects across samples were corrected using Harmony (v0.1.1). The first 15 significant principal components (PCs) were used for t-distributed stochastic neighbor embedding (t-SNE). The optimal number of PCs was determined using an Elbow Plot to visualize the cumulative variance explained by the principal components. The Elbow Plot helped identify the point where additional components contribute minimal variance, guiding the selection of the number of PCs used for subsequent analysis. Cell clustering was performed with resolution = 0.5, and cluster-specific DEGs were identified using Wilcoxon rank-sum testing. Marker genes were defined as those with adjusted *p* value < 0.05 and |log2FC| > 1.

### 2.4. Angiogenesis-Related Differential Genes and Their Visualization

The gene sets labeled as angiogenesis-related clusters (tumor vasculature, vascular smooth muscle cells (vSMCs), AVR (arteriovenous remodeling)-like vasculature) were intersected with bulk and scRNA-seq DEGs to obtain angiogenesis-related DEGs (angiogenesis-deg). Venn diagrams showed overlaps. Heatmaps visualized expression patterns.

### 2.5. Enrichment Analysis of Angiogenesis-Related Differential Genes

Kyoto Encyclopedia of Genes and Genomes (KEGG) and Gene Ontology (GO) enrichment analyses were performed using the clusterProfiler (v4.8.3) package. Fisher’s exact test was applied to assess term significance, and *p* values were adjusted using the Benjamini–Hochberg method. An adjusted *p* value < 0.05 indicated significant enrichment. The enrichment results were visualized using network plots.

### 2.6. Prognostic Assessment of Angiogenesis-Related Differential Genes

To improve robustness and reduce false positives, candidate genes were sequentially filtered by (i) differential expression evidence from bulk transcriptomic datasets (TCGA-KIRC and multiple GEO cohorts), (ii) restriction to angiogenesis-related vascular clusters derived from scRNA-seq (angiogenesis-deg), and (iii) stepwise survival modeling (univariate Cox regression → the least absolute shrinkage and selection operator (LASSO) → multivariable Cox regression). First, univariate Cox regression was applied to the TCGA-KIRC transcriptome data to identify survival-associated genes (adjusted *p* value < 0.05). These survival-related genes were then intersected with angiogenesis-deg to obtain angiogenesis-cox genes. LASSO regression was then applied to the angiogenesis Cox genes to reduce redundancy, followed by univariate Cox regression. Genes with *p* value < 0.05 were entered into a multivariable Cox regression model, and genes remaining significant in the multivariable model were defined as angiogenesis-hub genes. Patients were stratified into high- and low-risk groups using the median risk score. Kaplan–Meier (KM) curves were generated to compare OS between groups, and differences were assessed using the log-rank test.

Risk score formula:

$$Risk\;score=\sum_{i}^{n}Coef\left({gene}_{i}\right)\times Expression\left(gene_{i}\right)$$

where Coef (geneᵢ) represents the regression coefficient of gene i derived from the multivariate Cox model, Expression(geneᵢ) denotes the expression value of gene i, and n is the number of genes included in the model.

### 2.7. Analysis of Immune Infiltration and Immune Checkpoint Characteristics

#### 2.7.1. Estimation of Immune Cell Infiltration

Immune cell infiltration was evaluated using the CIBERSORTx algorithm. The algorithm infers the relative proportions of 22 immune cell subtypes based on gene expression profiles and performs 1000 permutations to enhance the robustness of the deconvolution. Only samples with a CIBERSORTx output *p* value < 0.05 were included to construct the final immune infiltration matrix.

#### 2.7.2. Correlation Analysis Between Risk Score and Immune Features

Spearman correlation analysis was conducted to assess the relationships between the risk score and immune cell infiltration levels, as well as immune checkpoint molecule expression, thereby characterizing the immune features associated with angiogenesis-related risk patterns.

#### 2.7.3. Correlation Analysis Between Angiogenesis-Hub Genes and Immune Features

Spearman correlation analysis was further applied to evaluate the associations between the expression of angiogenesis-hub genes and immune cell infiltration, as well as immune checkpoint expression, to elucidate the potential roles of angiogenesis-related genes within the tumor immune microenvironment.

### 2.8. Somatic Mutation Analysis

Somatic mutation data for TCGA-KIRC were retrieved using the R package TCGAbiolinks (v2.28.3). The data were processed into Mutation Annotation Format files and subsequently analyzed using the R package maftools (v2.16.0) to characterize the somatic mutational landscape.

### 2.9. Radiogenomics Workflow

#### 2.9.1. ROI Delineation and Radiomic Feature Extraction

Tumor three-dimensional regions of interest (ROIs) were manually delineated in 3D Slicer (v5.8.0) by two radiologists with more than ten years of experience. Radiomic feature extraction was performed using the SlicerRadiomics module (based on PyRadiomics v3.0.1). To mitigate variability due to scanner differences and slice thickness, all images were resampled to isotropic voxels (1 × 1 × 1 mm^3^) using spline interpolation. Gray-level intensities were discretized using a fixed bin width of 25 Hounsfield units, and intensity normalization was applied to ensure feature comparability. Extracted features included: first-order statistics, gray-level co-occurrence matrix (GLCM), gray-level dependence matrix (GLDM), gray-level run-length matrix (GLRLM), gray-level size zone matrix (GLSZM), neighboring gray tone difference matrix (NGTDM), wavelet-transformed features from eight sub-bands (LHL, LHH, LLH, HHL, HLH, HLL, LLL, HHH). All radiomic features were computed in accordance with the Image Biomarker Standardization Initiative [22].

#### 2.9.2. Feature Stability Assessment and Preprocessing

To assess reproducibility and robustness of radiomic features, ROIs of 20 randomly selected cases were re-delineated by the same reader after one month. The intraclass correlation coefficient (ICC) was calculated using a two-way random-effects model ICC. Features with ICC > 0.75 were considered stable and retained for further analysis. The selected features were standardized using Z-score normalization prior to model development [23,24].

#### 2.9.3. Selection of Radiogenomic Features and Score Calculation

The minimum redundancy maximum relevance (mRMR) method was first applied to reduce redundancy among stable radiomic features and identify those most closely related to the angiogenesis-associated risk score. Subsequently, Spearman correlation analysis was performed, and features with an absolute correlation coefficient |r| > 0.30 were retained. The Spearman correlation coefficients served as weighting factors for constructing the radiogenomics score according to the following formula:

$$\mathrm{Radiogenomics}\;\mathrm{score}\;=\sum_{i=1}^{n}r_{i}\times X_{i}$$

where $r_{i}$ denotes the Spearman correlation coefficient of feature *i*, $X_{i}$ denotes its standardized value, and n is the number of features included in the score.

#### 2.9.4. Construction and Validation of the ccRCC Radiogenomics Prognostic Model

The dataset was randomly split into training and validation cohorts at a 7:3 ratio using stratified sampling based on survival outcomes. The optimal cutoff value for the radiogenomics score was determined and used to divide patients into high- and low-score groups. Clinical characteristics were categorized as follows: Stage I–II (localized), Stage III (locally advanced), and Stage IV (metastatic); G1–G2 as low grade and G3–G4 as high grade.

A series of machine learning and statistical algorithms were employed to construct prognostic models for 1-, 3-, and 5-year OS, including Cox regression, LASSO, ridge regression, elastic net, decision tree, random forest, gradient boosting machine (GBM), extreme gradient boosting (XGBoost), and a stacked ensemble model (combining LASSO, decision tree, and random forest). All models were trained using 10-fold cross-validation to minimize overfitting. Model performance was assessed by time-dependent C-index, Brier score, and calibration curves. Decision curve analysis (DCA) was used to evaluate clinical utility. The SHAP (Shapley Additive Explanations) framework was applied to interpret model predictions and quantify feature contributions. The final model was deployed as an online clinical prediction tool via Shinyapps.io. All preprocessing and model selection (feature selection, hyperparameter tuning, and cutoff definition) were performed within cross-validation on the training set, and the validation/external cohorts were evaluated only after applying training-derived preprocessing parameters to avoid information leakage.

### 2.10. Analysis of the Biological Behavior of Single Genes and Their Correlation with Clinical Baseline Characteristics

To investigate the potential biological mechanisms associated with the gene of interest and its relationship with clinical baseline characteristics, single-gene-based Gene Set Enrichment Analysis (GSEA) and correlation analyses were performed. The expression level of the gene was dichotomized into high- and low-expression groups based on the median value. Single-gene GSEA was conducted using the clusterProfiler (v4.8.3) package, and enrichment results with an absolute normalized enrichment score (|NES|) > 1 and a false discovery rate (FDR) < 0.05 were considered significant.

For association analyses, non-parametric statistical tests were applied due to the non-normal distribution of clinical variables. For categorical variables, the Chi-square test or Fisher’s exact test was used. For continuous variables, the Wilcoxon rank-sum test (two groups) or Kruskal–Wallis test (multiple groups) was employed. All *p* values were adjusted for multiple testing using the BH method where applicable.

### 2.11. Single-Gene Biological Validation

#### 2.11.1. Tumor Tissue Samples and Cell Line Acquisition

Paired tumor and adjacent normal tissues were collected from four patients with ccRCC who underwent radical nephrectomy. All procedures were conducted in accordance with the Declaration of Helsinki and were approved by the Institutional Review Board of the Second Hospital of Lanzhou University. Written informed consent was obtained from all participants. Human renal proximal tubular epithelial cells (HK-2) and renal carcinoma cell lines (ACHN, 786-O, Caki-2, and A-498) were used in this study. All cell lines were obtained from the Gansu Provincial Key Laboratory of Urological Diseases.

#### 2.11.2. Cell Line Culture Conditions

HK-2 cells were cultured in DMEM/F12 medium (Gibco, Grand Island, NY, USA) supplemented with 10% fetal bovine serum (PAN-Biotech, Aidenbach, Germany). 786-O cells were maintained in RPMI-1640 medium (Gibco, Grand Island, NY, USA) with 10% fetal bovine serum. Caki-2 cells were cultured in McCoy’s 5A medium (Basal Media, Heidelberg, Germany) with 10% fetal bovine serum. ACHN and A-498 cells were grown in MEM medium (Basal Media, Heidelberg, Germany) supplemented with 10% fetal bovine serum. HUVECs were cultured in Endothelial Cell Medium (ECM medium, Scien Cell, Carlsbad, CA, USA) with 10% fetal bovine serum (Scien Cell, Carlsbad, CA, USA). All cell lines were maintained in a humidified incubator at 37 °C with 5% CO_2_.

#### 2.11.3. Western Blotting

Total proteins were extracted from tumor tissues, paired adjacent tissues, and cell lines. Equal amounts of protein (20–40 μg per lane) were separated using 10% SDS-PAGE and transferred onto 0.45 μm PVDF membranes (Millipore, Billerica, MA, USA). Membranes were blocked with Blocking Buffer (Beyotime, Shanghai, China, P0023B) for 30 min at room temperature and washed three times with TBST. Membranes were incubated overnight at 4 °C with primary antibodies against PDLIM1 (rabbit, 1:8000, Proteintech, Rosemont, IL, USA, 11674-1-AP) and β-actin (rabbit, 1:12,000, ABclonal, Wuhan, China, AC026). On the following day, membranes were incubated with DyLight 800-conjugated goat anti-rabbit secondary antibody (1:20,000, Abbkine, Wuhan, China, A23920) at room temperature, protected from light. After washing, protein bands were visualized using the Odyssey^®^ CLX Infrared Imaging System (LI-COR Biosciences, Lincoln, NE, USA). Densitometric quantification was performed using ImageJ2 (v2.14), and protein expression levels were normalized to β-actin.

#### 2.11.4. Generation of Stable Cell Lines

Stable cell lines were established by lentiviral transduction. A PDLIM1 overexpression vector was introduced into 786-O cells, while two independent Short Hairpin RNA (shRNA-targeting) PDLIM1 (shPDLIM1#1 and shPDLIM1#2) were used to generate knockdown lines in A-498 cells. Lentivirus construction and packaging were outsourced to Tsingke Biotechnology (Beijing, China). After antibiotic selection, stable populations were maintained, and PDLIM1 expression was confirmed by Western blotting.

#### 2.11.5. Conditioned Medium Generation

To prepare conditioned medium (CM) for endothelial cell-related assays, stable 786-O (PDLIM1-OE and matched NC) and A-498 (shNC, shPDLIM1#1, and shPDLIM1#2) cells were cultured in their respective complete media until reaching approximately 80% confluence. The medium was then replaced with a mixed medium containing ECM medium. Specifically, 786-O cells were incubated in 50% RPMI-1640 and 50% ECM medium, whereas A-498 cells were incubated in 50% MEM and 50% ECM medium. After incubation for 48 h, the supernatants were collected, cleared by centrifugation, and filtered through a 0.22 μm filter (Servicebio, Wuhan, China, SF-PES-223301). Filtered CM was aliquoted and stored at −80 °C until use.

#### 2.11.6. Tube Formation Assay

For tube formation, 24-well plates (Labselect, Beijing, China, 11310) were coated with 20 μL Matrigel (Corning, Corning, NY, USA, 356234) per well and incubated at 37 °C until the gel solidified. HUVECs were cultured in the indicated CM for 48 h and then seeded onto the Matrigel-coated wells at a density of 1 × 10^5^ cells per well. After incubation for 6 h, tube-like structures were examined under a light microscope (Olympus, Tokyo, Japan) and images were captured for subsequent quantification.

### 2.12. Statistical Analysis

All statistical analyses were performed using R software (v4.3.0) and GraphPad Prism (v9.0). Unless otherwise stated, two-sided tests were used. Statistical significance was defined as *p* value < 0.05 or adjusted *p* value < 0.05, with multiple testing correction performed using the Benjamini–Hochberg method where applicable.

## 3. Results

### 3.1. Differential Gene Expression Data from Transcriptome

Transcriptome data from seven microarrays were aggregated to classify benign and malignant samples, annotate genes, and remove batch effects, resulting in a total of 11,789 genes sourced from 219 normal tissues and 297 ccRCC tissues (Supplementary Figure S2A,B). After batch correction and differential analysis using limma with a Benjamini–Hochberg adjustment, 1493 differential genes (GEO-deg) were identified (Supplementary Figure S2C). For the TCGA dataset, transcriptomic data from 72 normal tissues and 541 tumor tissues were processed, yielding 60,533 genes (Supplementary Figure S3A). Genes expressed in fewer than half of the samples were excluded, leaving 32,934 genes in the final dataset (Supplementary Figure S3B). Differential expression analyses using limma, DESeq2, and edgeR were performed, and the intersection of DEGs identified by all three algorithms yielded 7614 differential genes (TCGA-deg; Supplementary Figure S3C,D).

From the single-cell transcriptome data, 5 ccRCC samples (pathological stage T3a) were obtained, and the filtering process for the single-cell transcriptome matrix is detailed in Supplementary Figure S4. scRNA-seq analysis identified major immune, stromal/vascular, and tumor cell populations (Supplementary Figure S5), and cluster annotation followed the original publication [25]. Differential gene analysis across these 15 clusters yielded a set of single-cell differential genes (scRNA-deg). The gene counts per cell type, as well as the proportion of each cell type in every sample, are shown in Supplementary Figure S5B,C. The most representative marker genes for each cell type are illustrated in Supplementary Figure S5D, and the top 5 marker genes per cell type are listed in Supplementary Figure S5E.

### 3.2. Differential Genes and Enrichment Analysis in Clear Cell Renal Carcinoma

**Figure 2:**
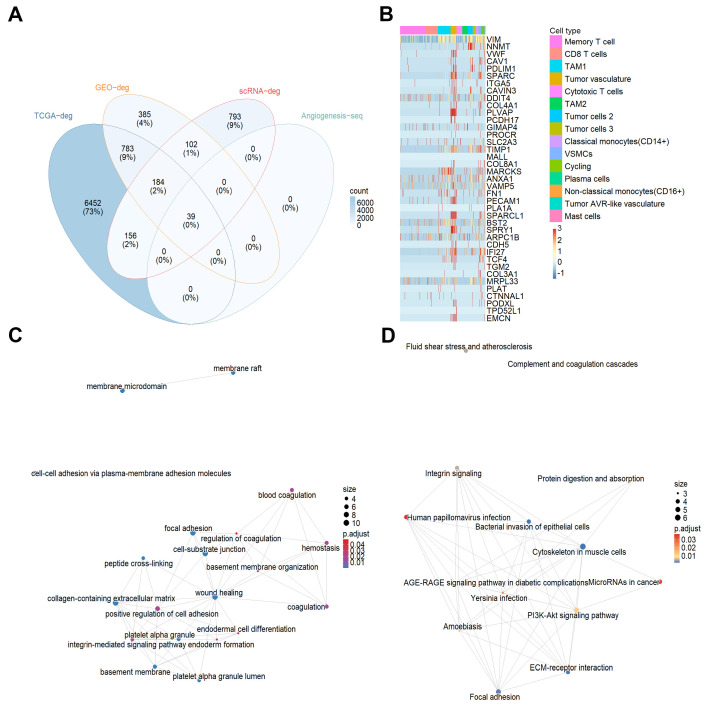
Visualization of angiogenesis-deg and their enrichment analysis. (**A**) Venn diagram of angiogenesis-deg. (**B**) Heatmap of the distribution of angiogenesis-deg in each cell group. (**C**) GO enrichment analysis network diagram of angiogenesis differential genes. (**D**) KEGG enrichment analysis diagram of angiogenesis-deg. TAM, tumor-associated macrophages. vSMCs, vascular smooth muscle cells.

The intersection of differential genes from TCGA, GEO, and single-cell RNA sequencing data was visualized using a Venn diagram, and these genes were further intersected with gene sets related to vSMCs, tumor vasculature, and tumor AVR-like vasculature, resulting in 39 angiogenesis-related differential genes (angiogenesis-deg) (Figure 2A). The heatmap in Figure 2B illustrates the distribution of angiogenesis-deg across various cell types. The functional enrichment network constructed from GO and KEGG analyses demonstrated that angiogenesis-deg mainly converged into four functional clusters (Figure 2C,D). The most prominent category was related to cell adhesion and extracellular matrix (ECM) organization, including pathways such as “Focal adhesion” and “ECM-receptor interaction.” Another cluster was centered around coagulation and hemostasis, with the “Complement and coagulation cascades” pathway prominently featured. Other clusters involved membrane domains (e.g., “membrane raft”) and vascular pathologies (e.g., “Fluid shear stress and atherosclerosis,” and “advanced glycation end products–receptor for advanced glycation end products (AGE-RAGE) signaling pathway in diabetic complications”). The enrichment network highlights “Focal adhesion” and “ECM-receptor interaction” as key pathways, underscoring the importance of cell-ECM interactions in the functional profile of angiogenesis-deg. Further detailed information is available in Supplementary Figure S6.

### 3.3. Prognostic Analysis of Angiogenesis-Related Differential Genes

**Figure 3:**
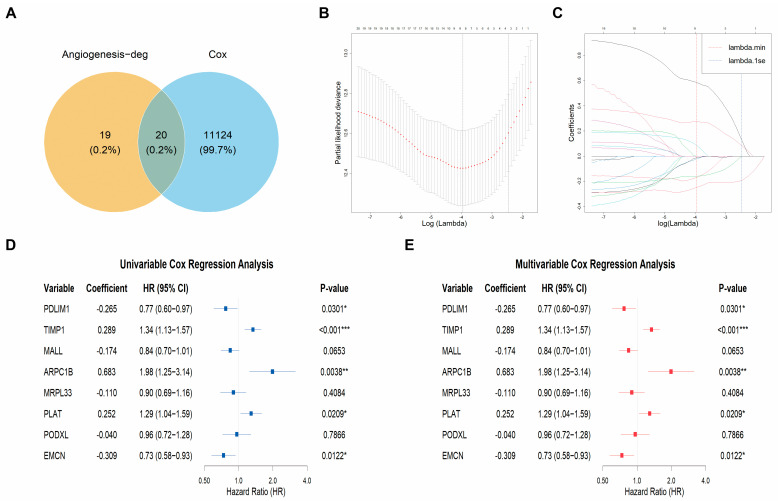
Selection process of angiogenesis-cox genes and angiogenesis-hub genes. (**A**) Venn diagram of angiogenesis-cox genes. (**B**) LASSO algorithm was employed to select angiogenesis-hub genes, confirming the optimal tuning parameter (λ) through 5-fold cross-validation, with λ min = 0.012 and log(λ_min_) = −4.43. (**C**) Plot of LASSO coefficients versus log(λ), where 8 genes exhibiting the lowest LASSO coefficients were identified at log(λ_min_) = −4.43. (**D**,**E**) Univariate and multivariate Cox regression analyses further refined the selection of angiogenesis hub genes. CI: confidence interval. HR: hazard ratio. LASSO: the least absolute shrinkage and selection operator. * *p* value < 0.05; ** *p* value < 0.01; *** *p* value < 0.001.

**Figure 4:**
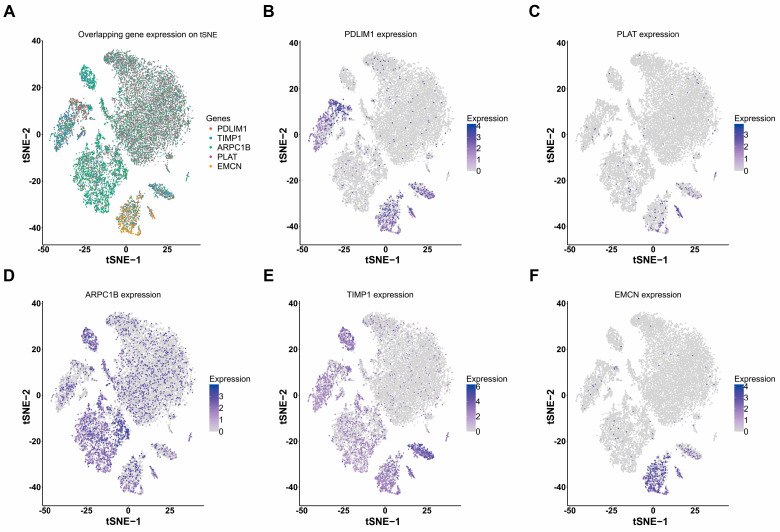
The tSNE plot of angiogenesis-hub genes. (**A**) The overall distribution pattern of angiogenesis-hub genes on the tSNE plot. (**B**–**F**) The respective cell distribution of angiogenesis-hub genes on the tSNE plot.

After removing duplicates from the TCGA tumor transcriptome dataset, we obtained OS data for 513 ccRCC samples. Initially, 32,934 genes were filtered to retain those with expression levels greater than 10 in more than half of the samples, resulting in 20,083 genes. Cox regression analysis revealed 11,144 genes significantly associated with OS. A total of 20 angiogenesis-cox genes were identified (Figure 3A). Subsequently, LASSO regression was used to select 8 genes, which were then evaluated using univariate Cox regression analysis. Genes with *p* values less than 0.05 were selected and further included in multivariate Cox regression analysis. Finally, 5 angiogenesis-related hub genes were identified: PDLIM1, EMCN, ARPC1B, PLAT, and TIMP1 (Figure 3B–E and Figure 4A–E). The cellular distribution of these angiogenesis-hub genes is presented in Figure 4. The risk score for the gene set was calculated using the following formula:

Risk score = (−0.351 × PDLIM1) + (−0.388 × EMCN) + (0.508 × ARPC1B) + (0.243 × PLAT) + (0.295 × TIMP1).

A binary classification was performed based on the median expression levels of each gene to assess the correlation between high- and low-expression groups concerning OS. KM survival curves were generated for each gene (Supplementary Figure S7A–E). Additionally, ccRCC patients were stratified into high-risk and low-risk groups based on their risk scores, and a significant difference in OS was observed between the groups (Supplementary Figure S7F–H). The heatmap showing the expression levels of PDLIM1, EMCN, ARPC1B, PLAT, and TIMP1 in the two groups is shown in Supplementary Figure S7I. Together, these analyses defined a five-gene angiogenesis-related signature that stratified patients into distinct survival risk groups.

### 3.4. Relationship Between Angiogenesis-Hub Genes, Risk Score and Tumor Immune Microenvironment, Somatic Mutations

Analysis of the tumor immune microenvironment revealed that increased levels of immune cells, such as resting natural killer cells, monocytes, M1 macrophages, dendritic cells, resting mast cells, and eosinophils, were significantly associated with improved patient survival (Supplementary Figure S8). Furthermore, high-risk scores were associated with increased sensitivity to immune checkpoint genes such as cytotoxic T-lymphocyte–associated protein 4 (CTLA4), lymphocyte activation gene 3 (LAG3), and CD276, whereas low-risk scores showed higher sensitivity to NRP1, CD274 (PD-L1), and TNFRSF4 (Supplementary Figure S9A).

Somatic mutation profiling in TCGA-KIRC showed that VHL, PBRM1, TTN, SETD2, and BAP1 were the most frequently mutated genes, with VHL, PBRM1, and TTN ranking among the top alterations in both risk groups (Supplementary Figure S10).

### 3.5. Radiomics Features

A total of 199 ccRCC patients from TCIA-KIRC with available contrast-enhanced CT images and matched transcriptomic data were included in the radiomics analysis, divided into a training set (139 patients) and an internal validation set (60 patients). ROIs were delineated for each lesion, and 837 radiomic features were initially extracted. After screening for reproducibility using the ICC, 811 standardized features were retained for further analysis (Supplementary Figure S11). The mRMR algorithm reduced this feature set to 20 features, and Spearman correlation analysis identified 6 radiomics features with significant relevance, from which their corresponding radiogenomics scores were calculated (Supplementary Figure S12).

Radiogenomics score = (0.23 × gldm_wavelet_HHL_DependenceNonUniformityNormalized) + (0.21 × glrlm_wavelet_LLH_RunVariance) + (0.21 × glrlm_wavelet_LLL_RunVariance) + (−0.22 × firstorder_wavelet_HLL_Skewness) + (−0.23×ngtdm_original_Strength) + (−0.28 × glszm_wavelet_LLL_SmallAreaEmphasis)

### 3.6. Construction of the Radiogenomics Prognostic Model

Tumor Stage was identified as the most important clinical baseline characteristic for constructing the prediction model (Table 1). The combined prediction model for OS in ccRCC patients was constructed using stage and radiogenomics scores. The optimal cut-off value for the radiogenomics score was determined as −0.0759 based on the Youden index in the training cohort, and patients were stratified into high-score and low-score groups. KM curve analysis indicated that higher stage and higher risk scores were associated with poorer prognosis in ccRCC (all *p* values < 0.05) (Supplementary Figure S13).

**Figure 5:**
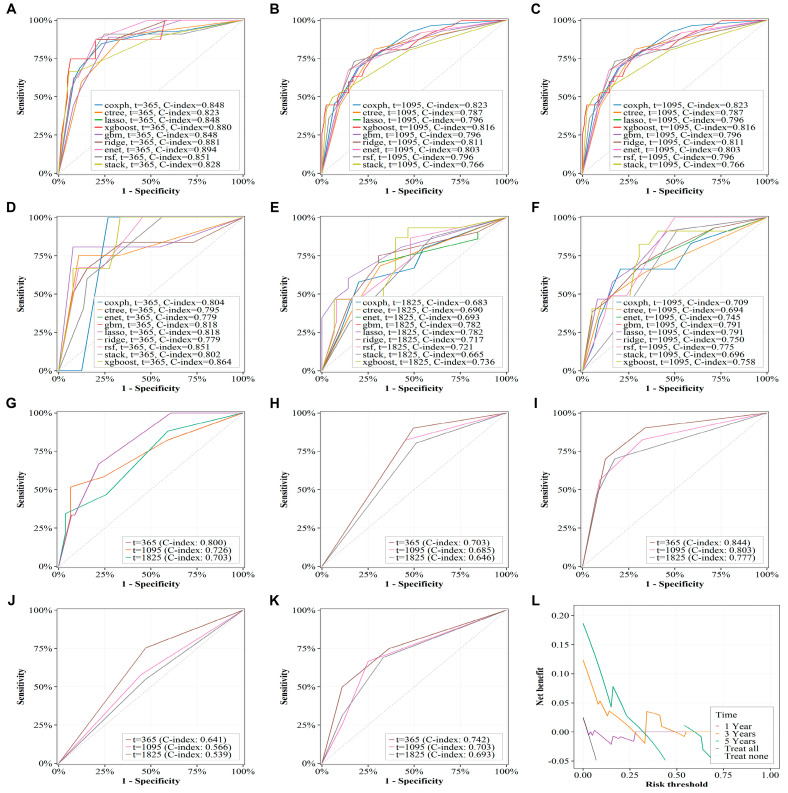
Time-dependent C-index and decision curve of predictive models developed using various algorithms. (**A**–**F**) C-index of multiple algorithms for predicting 1-, 3-, and 5-year overall survival in ccRCC patients across the training (**A**–**C**) and internal validation (**D**–**F**) sets. (**G**) C-index for 1-, 3-, and 5-year overall survival in the external validation set. (**H**,**I**). C-index of the training set for the XGBoost models developed based on Stage and radiogenomics score respectively. (**J**,**K**) C-index of the internal validation set for the xgboost models developed based on Stage and radiogenomics score respectively. (**L**) Decision curve analysis of the XGBoost prediction model on the external validation set. Coxph: Cox regression. Ctree: Decision tree. Lasso: Least absolute shrinkage and selection operator regression. Xgboost: extreme gradient boosting. Gbm: Gradient boosting machine. Ridge: Ridge regression. Enet: Elastic net. Rsf: Random forest. Stack: Stacked machine learning. CI: Confidence interval.

The XGBoost-based combined model showed the best overall performance among evaluated algorithms (Supplementary Tables S1 and S2), with consistent discrimination in the training and internal validation sets (Table 2; Figure 5A–F). In external validation (N = 121, Supplementary Table S3), performance remained stable, with time-dependent C-indices of 0.800, 0.726, and 0.703 at 1, 3, and 5 years, respectively (Table 2; Figure 5G). The DCA supported good agreement between predicted and observed risk and demonstrated favorable net benefit for 1-, 3-, and 5-year OS prediction in the evaluated cohorts (Figure 5L; Supplementary Figure S14).

In the radiogenomics model, the radiogenomics score provided prognostic information complementary to tumor stage, and the combined model consistently achieved higher discrimination than stage alone across internal and external validation sets.

**Figure 6:**
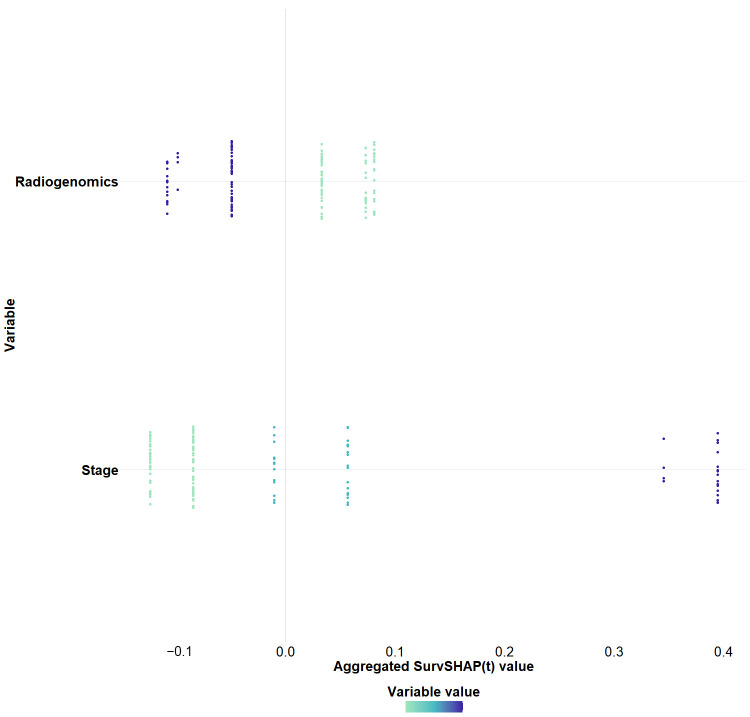
Visualization of the XGBoost predictive model based on SHAP values. The color changes from light to dark, representing the gradual increase in the variable value.

Visualization of XGBoost model results through SHAP values showed that the radiogenomics score was the most important factor in the model. The stage and radiogenomics scores all had a positive impact on predicting OS in ccRCC patients (Figure 6). The web address of the online prediction model developed using XGBoost is: https://aa-ccrcc-prediction-model.shinyapps.io/shinydashboard_sa_xgboost/ (accessed on 15 Janurary 2026).

In practical terms, a C-index of ~0.70–0.80 indicates moderate-to-good discrimination for ranking patients by mortality risk. Using the training-derived radiogenomics score cutoff derived in the training cohort, the combined model more consistently separates higher- from lower-risk patients than stage alone across validation cohorts. This type of risk stratification could support postoperative counseling and risk-adapted surveillance intensity, although prospective decision-impact evaluation is required before clinical adoption.

### 3.7. Biological Behavior of PDLIM1 and Its Relationship with Clinical Baseline Characteristics

Current research on PDLIM1 in ccRCC is limited, as evidenced by the literature review and correlation analysis results. Consequently, PDLIM1 was selected for further investigation. The correlation analysis revealed that, compared to normal tissues, the mRNA levels of PDLIM1 were elevated in tumor tissues (Supplementary Figure S15A). Significant differences in PDLIM1 mRNA levels were observed between T staging T1 and T4, as well as between T3 and T4, with higher T staging correlating with lower expression. However, no significant differences were found between the N and M staging. In the age group >60, PDLIM1 expression was lower compared to the ≤60 age group, with a significant difference between the two groups. Similarly, expression differences were observed between grades G1 and G4, as well as G2 and G4, with expression decreasing as the grade increased. Moreover, PDLIM1 expression differed between Stage I and Stage II, with higher stages associated with lower expression. No differences were observed in PDLIM1 expression based on gender or race (Supplementary Figure S15B–I).

**Figure 7:**
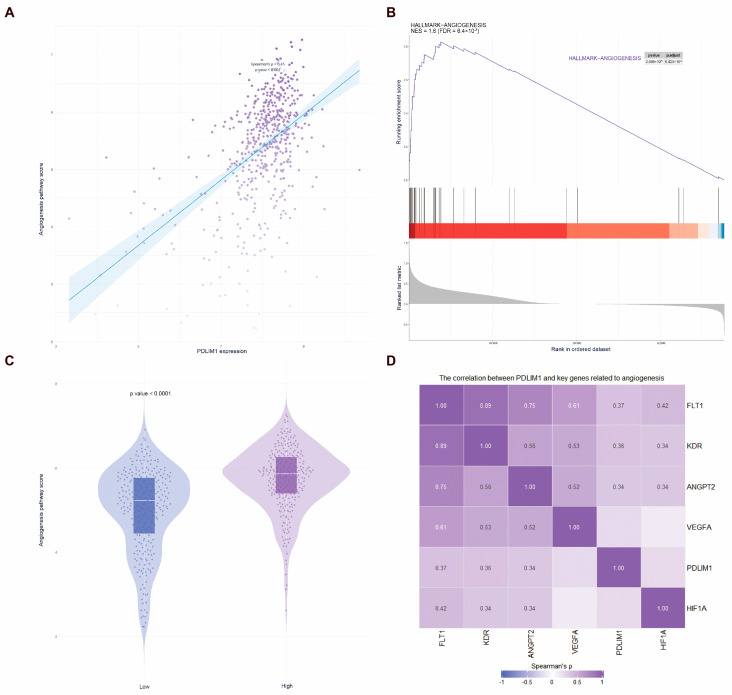
Correlation analysis of PDLIM1 with angiogenesis. (**A**). Correlation analysis of PDLIM1 and angiogenesis pathway scores. (**B**) Gene Set Enrichment Analysis of PDLIM1; the color bars indicate the direction and intensity of the correlation with the grouping (PDLIM1 high/low expression group) (red: positive correlation/high expression group enrichment; blue: negative correlation/low expression group enrichment). (**C**) Differences in angiogenesis scores between high- and low-PDLIM1-expression groups. (**D**) Correlation analysis of PDLIM1 with angiogenesis genes.

Single-gene enrichment analysis of PDLIM1 indicated that it is primarily involved in angiogenesis, hypoxia, and epithelial–mesenchymal transition (Supplementary Figure S16). PDLIM1 showed a positive correlation with angiogenesis pathway scores, calculated based on the expression of VEGFA, ANGPT1, ANGPT2, PDGFB, and TIE1 (Figure 7A), and plays a promotive role in vascular pathway regulation (Figure 7B). Significant differences in angiogenesis pathway scores were observed between the low- and high-PDLIM1-expression groups, with higher expression correlating with stronger angiogenesis scores (Figure 7C). We also examined the correlation between PDLIM1 expression and key angiogenesis genes. Strong correlations were found between PDLIM1 and VEGF receptors (FLT1, KDR) and ANGPT2, suggesting that PDLIM1 may contribute to angiogenesis and remodeling. However, PDLIM1 exhibited weaker correlations with VEGFA and HIF1A, indicating that its regulation of angiogenesis may not depend on VEGFA expression or the classical hypoxia signaling pathway (Figure 7D).

### 3.8. Validation of PDLIM1 Expression in Human Tissues and Cell Lines

**Figure 8:**
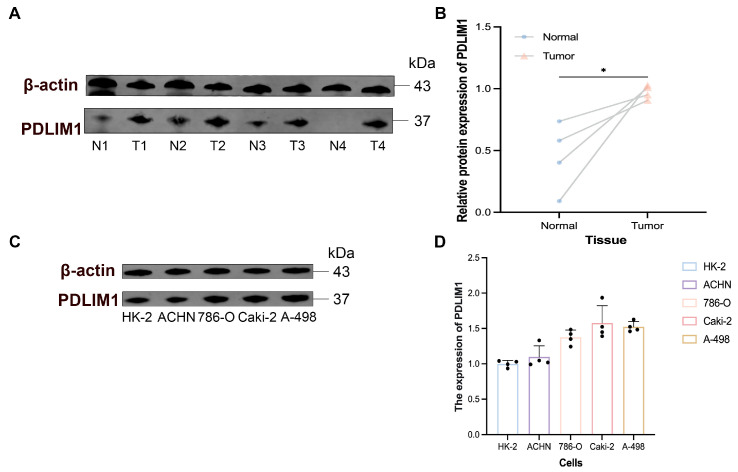
Protein expression levels of PDLIM1. (**A**,**B**) Protein expression levels of PDLIM1 in four pairs of normal and cancer tissues. (**C**,**D**) Protein expression levels of PDLIM1 in different cell lines. Densitometry was performed in ImageJ; band intensities were normalized to β-actin and expressed relative to the mean normalized value of the HK-2 group. N: normal. T: tumor. * *p* value < 0.05.

Analysis using The Cancer Cell Line Encyclopedia (CCLE) database revealed that PDLIM1 mRNA expression was highest in the SNU267 cell line and lowest in the G402 cell line (Supplementary Figure S17). Quantitative protein analysis of 4 paired tumor and adjacent normal tissues from our research center showed that PDLIM1 protein expression was elevated in tumor tissues compared to adjacent normal tissues (Figure 8A,B). The raw blots are shown in Supplementary Figure S18. Furthermore, we validated PDLIM1 expression in several cell lines (Figure 8C), and the corresponding raw blots are provided in Supplementary Figure S19. Compared to the HK-2 cell line, PDLIM1 protein expression was significantly higher in the 786-O, Caki-2, and A-498 cell lines, with statistically significant differences (Figure 8D).

### 3.9. PDLIM1 Stable Cell Lines Were Successfully Established and Validated

**Figure 9:**
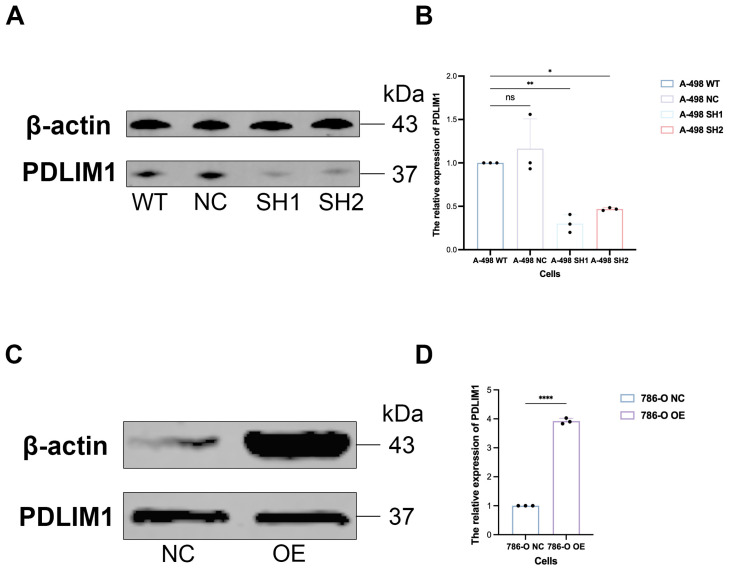
Establishment and validation of stable PDLIM1 overexpression and knockdown cell lines. (**A**,**B**) knockdown of PDLIM1 in A-498 cells; (**C**,**D**) Overexpression of PDLIM1 in 786-O cells. WT, wild-type cells; NC, Negative control (cells transduced with empty vector); OE, PDLIM1 overexpression; SH1/SH2: PDLIM1 knockdown by shRNA 1/shRNA 2 (stable cell lines); * *p* value < 0.05; ** *p* value < 0.01; **** *p* value < 0.0001.

To investigate the role of PDLIM1 in tumor cell-driven angiogenic activity, stable cell models were generated. PDLIM1 was stably overexpressed in 786-O cells, and two independent shRNAs (SH1 and SH2) were used to establish PDLIM1 knockdown in A-498 cells. Western blotting confirmed robust PDLIM1 OE in 786-O cells and efficient PDLIM1 depletion in A-498 SH1/SH2 cells compared with their respective negative controls (Figure 9), and the corresponding raw blots are provided in Supplementary Figures S20 and S21.

### 3.10. Tumor Cell-Derived Conditioned Medium Regulated HUVEC Tube Formation in a PDLIM1-Dependent Manner

**Figure 10:**
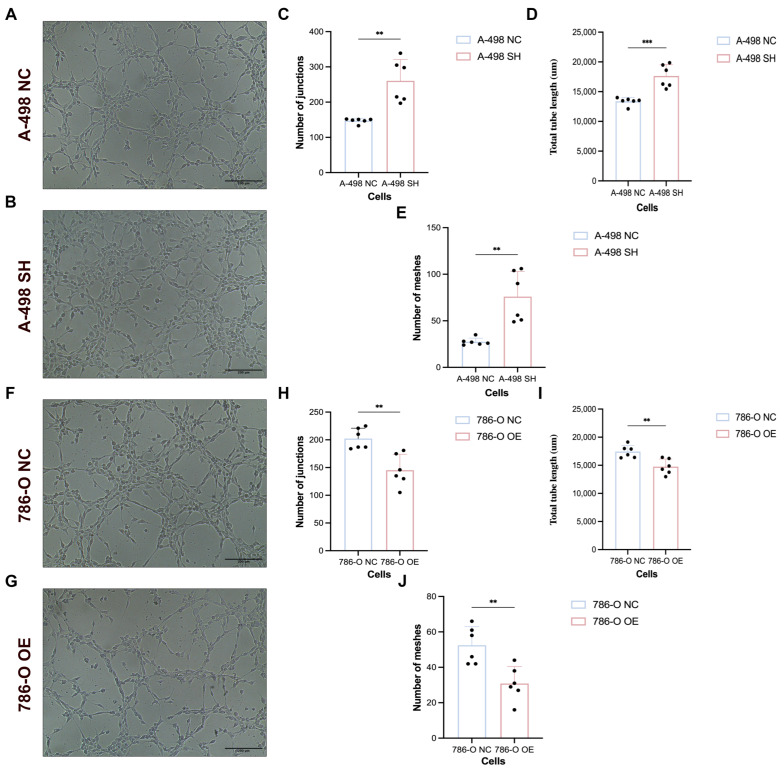
PDLIM1 suppresses tumor cell-derived CM-induced HUVEC tube formation. (**A**,**B**) Representative images of HUVEC tube formation following exposure to conditioned medium (CM) from A-498-NC or A-498-SH cells; (**C**–**E**) Quantification of tube formation in the A-498-CM groups, including number of junctions (**C**), total tube length (**D**), and number of meshes (**E**); (**F**,**G**) Representative images of HUVEC tube formation following exposure to CM from 786-O-NC or 786-O-OE cells; (**H**–**J**) Quantification of tube formation in the 786-O-CM groups, including number of junctions (**H**), total tube length (**I**), and number of meshes (**J**). NC, negative control; SH, PDLIM1 knockdown; OE, PDLIM1 overexpression; **p value < 0.01; ***p value < 0.001.

To assess whether PDLIM1 affects paracrine pro-angiogenic signaling, CM were collected from stable 786-O and A-498 cell lines and applied to HUVECs prior to tube formation assays. HUVECs pretreated with CM from PDLIM1OE formed less extensive capillary-like networks on Matrigel than those treated with CM from 786-O NC cells, as evidenced by reduced tube complexity on representative images. In contrast, CM derived from A-498 SH (SH1) cells promoted enhanced tube formation compared with CM from A-498 NC cells. Quantification of tube formation further supported that altering PDLIM1 expression in tumor cells modulates the angiogenic behavior of HUVECs through soluble factors (Figure 10). Collectively, these results support that tumor-cell PDLIM1 modulates endothelial tube formation through soluble factors, consistent with an effect on tumor-driven angiogenic signaling in vitro.

## 4. Discussion

ccRCC is one of the most common malignancies in the urinary system, and even after radical nephrectomy, metastasis can still occur. Moreover, metastatic ccRCC is resistant to both local radiation therapy and systemic chemotherapy, leading to a 5-year survival rate of only 11.7% [26]. The majority of ccRCC cases are characterized by VHL gene inactivation, resulting in the sustained activation of HIF2α, which drives the expression of angiogenic factors and promotes tumor angiogenesis, a process associated with the prognosis of ccRCC [4]. Therefore, identifying novel angiogenesis-related prognostic markers is crucial.

In this study, we integrated bulk transcriptomic (TCGA-KIRC and GEO), single-cell RNA-seq, and radiomic features to identify angiogenesis-related biomarkers and construct an imaging–molecular prognostic framework for ccRCC. In external validation, the combined radiogenomics model demonstrated consistent discrimination and calibration, supporting the premise that linking CT radiomic phenotypes to angiogenesis-related transcriptomic risk programs can provide prognostic information complementary to conventional clinicopathological stratification and may capture aspects of intratumoral heterogeneity that are difficult to assess by biopsy or routine pathology alone.

In ccRCC, several prognostic models have been proposed to estimate postoperative outcomes, including clinicopathological risk scores such as the Leibovich, Stage, Size, Grade, and Necrosis score (SSIGN), and UCLA Integrated Staging System (UISS) [27,28,29,30,31,32]. Although these tools are clinically useful, their performance is constrained by reliance on clinicopathological variables, which may not fully capture intratumoral heterogeneity or pathway-level tumor biology [33]. Molecular profiling can provide mechanistic insight and support precision treatment planning [34]; however, tissue-based sequencing is limited by invasiveness, cost, and sampling bias in the presence of spatial heterogeneity [35,36]. Radiomics offers a noninvasive way to characterize whole-tumor imaging phenotypes and has been increasingly combined with clinical variables to build prognostic models in ccRCC [37,38,39,40]. Nevertheless, radiomics-only signatures can be difficult to interpret biologically and may be sensitive to scanner- and protocol-related variability, which can limit portability across institutions. By contrast, radiogenomics explicitly links imaging phenotypes to molecular programs. In our study, anchoring radiomic patterns to angiogenesis-related biomarkers derived from cross-cohort bulk transcriptomics and scRNA-seq constraints improves biological interpretability and reduces the likelihood of purely data-driven, unstable imaging signatures. Our model achieved consistent discrimination across cohorts while explicitly anchoring radiomic patterns to angiogenesis-related transcriptomic risk states, which may enhance interpretability and translational relevance. Nevertheless, radiogenomic robustness still depends on standardized imaging acquisition and reproducible segmentation; thus, multicenter harmonization and prospective validation remain essential.

We identified five angiogenesis-related prognostic biomarkers (PDLIM1, EMCN, ARPC1B, PLAT, and TIMP1). High expression of PDLIM1, EMCN, and PLAT was associated with favorable patient survival, whereas elevated levels of ARPC1B and TIMP1 indicated a poor prognosis. This finding regarding TIMP1 is consistent with a previous study in gastric cancer, where its high expression was also linked to poorer outcomes, suggesting a potential conserved oncogenic role across cancer types [41]. Similarly, the adverse prognostic effect of ARPC1B in our study aligns with its reported function in ccRCC, where it promotes tumor growth and invasion by activating the Wnt/β-catenin signaling pathway and inducing epithelial–mesenchymal transition [42]. Conversely, the protective role of EMCN is supported by functional evidence showing it is down-regulated in ccRCC, and its knockdown inhibits cancer cell proliferation [43]. PLAT, a key component of fibrinolysis, may influence extracellular matrix remodeling and tumor dissemination, which could partly explain its favorable prognostic association in our cohort.

Among these biomarkers, although PDLIM1 protein levels are upregulated in tumor tissues and ccRCC cell lines, it is associated with better survival rates. This pattern may seem counterintuitive, as tumor upregulation is typically interpreted as oncogenic. However, differential expression does not necessarily define functional directionality. Importantly, our functional assay results support a protective, anti-angiogenic role for PDLIM1 in tumor-endothelial cell interactions. Through CM experiments, CM from PDLIM1-overexpressing tumor cells inhibited HUVEC tube formation, while CM from PDLIM1 knockdown tumor cells enhanced tube formation, as reflected in total tube length, junctions, and meshes. These results suggest that PDLIM1 may suppress angiogenic activity through a paracrine mechanism, possibly by altering the secretion profile of soluble angiogenesis-related factors. This functional validation strengthens the interpretation of PDLIM1 as a favorable biomarker and provides biological rationale for its association with improved survival rates.

PDLIM1 (also known as CLP36/Elfin/CLIM1) is a cytoskeleton-associated PDZ-LIM family protein involved in actin-related organization and cellular adhesion. Dysregulated PDLIM1 has been reported in multiple cancers, including colorectal cancer [44], hepatocellular carcinoma [45], breast cancer [46], and gliomas [47], with context-dependent roles. Research has demonstrated that in colorectal cancer, PDLIM1 promotes the binding of β-catenin to E-cadherin, inhibiting tumor progression driven by the Wnt/β-catenin pathway [48]. In hepatocellular carcinoma, PDLIM1 binds to ACTN4, disrupting the interaction between ACTN4 and F-actin, which activates the Hippo pathway and inhibits tumor metastasis [45]. Moreover, PDLIM1 plays a role in regulating various mechanisms across different cancer tissues. This protein regulates several biological processes, including dynamic cytoskeletal remodeling and synaptic structure formation [49]. Existing studies suggest that PDLIM1 may regulate the progression of glioblastoma stem cells via the PI3K-AKT pathway [50]. PDLIM1 also modulates the Hippo pathway by inhibiting the E3 ligase AIP-4, which mediates YAP1 degradation, thereby promoting p53-deficient sarcoma [51]. Additionally, PDLIM1 directly regulates the expression of Wnt3a, influencing cell migration and invasion in diabetic retinopathy [52]. Consistent with this context-specific behavior, our data indicate that PDLIM1 in ccRCC is linked to favorable outcomes and may attenuate tumor-driven angiogenic signaling. Mechanistically, our bioinformatic analyses showed that PDLIM1 correlated positively with angiogenesis pathway scores and angiogenesis-related molecules such as ANGPT2 and VEGF receptors (FLT1/KDR), while correlations with core hypoxia-pathway genes (e.g., VEGFA and HIF1A) were weaker. Together with the CM-based functional results, these findings raise the possibility that PDLIM1 modulates angiogenesis through non-classical, hypoxia-independent routes, which warrants further mechanistic investigation.

Immunotherapy has fundamentally transformed the landscape of tumor treatment by demonstrating significant clinical efficacy across multiple malignancies [53]. Common immune checkpoint inhibitors include drugs targeting programmed death protein-1 (PD-1) (nivolumab, pembrolizumab, cemiplimab, and dostarlimab), programmed death-ligand 1 (PD-L1) (atezolizumab, avelumab, and durvalumab), CTLA-4 (ipilimumab and trelimumab), and LAG-3 (relatlimab) [53]. Unlike classical immune checkpoints, in our study, PDLIM1 shows a significant positive correlation with the expression of neurophilin-1 (NRP1) (Spearman ρ = 0.66, *p* value < 0.001). Neuropilins (NRPs) are members of a class of non-tyrosine kinase cell surface glycoproteins, with two subtypes, NRP1 and NRP2, primarily involved in various physiological and pathological processes as 120–140 kDa type I transmembrane proteins [54]. NRPs mainly function as class III signaling proteins and co-receptors of the VEGF family, drawing significant attention as potential targets for cancer therapy. These receptors play a crucial role in various cellular processes, including angiogenesis [55,56], cell survival [57], migration [58], and invasion [59], all of which are critical for tumor growth and metastasis [60]. NRP1 interacts with multiple ligands, such as vascular endothelial growth factor, semaphorins, and transforming growth factor-β, mediating various signaling pathways that promote tumorigenesis [61]. Overexpression of NRP1 has been observed in many cancer types and is associated with poor prognosis and increased tumor aggressiveness [62].

In the immune microenvironment, NRP1 primarily regulates the balance of immune cells in an indirect manner to exert immune functions. Treg cells play a crucial role in blocking autoimmunity, limiting immune pathology, and maintaining immune homeostasis in the immune system. Studies have shown that Treg cells maintain stability through the NRP1-Sema4a axis (enhancing resting/survival factors while inhibiting differentiation), and high expression of NRP1 is associated with inhibitory markers such as FOXP3 and GITR, facilitating tumor immune evasion. Conversely, the loss of NRP1 disrupts Treg function (e.g., reducing Akt phosphorylation and increasing Foxo3a nuclear localization), restoring anti-tumor immunity [63]. In CD8+ T cells, high expression of NRP1 is correlated with a reduction in the CD8+ T cell memory pool and poor response to immune checkpoint blockade therapy; targeting NRP1 may enhance persistent anti-tumor immunity [64]. TAMs act as tumor-promoting factors and immunosuppressive agents, as they can promote tumorigenesis by expressing cell surface receptors, secreting cytokines, chemokines, and enzymes that regulate the recruitment and functions of Tregs [65]. They serve as core drivers of the immunosuppressive tumor microenvironment. The deletion of NRP1 in macrophages inhibits TAMs from entering tumors in hypoxic environments [66].

In our study, we observed a significant correlation between PDLIM1 and NRP1, suggesting that both may potentially play a synergistic role in immune evasion. However, this remains a hypothesis that requires further experimental validation. While our prognostic analysis indicates that PDLIM1 associated with favorable outcomes in ccRCC, its association with the known immune checkpoint NRP1 may reflect complex microenvironment programs, which might contribute to immune evasion. Future research should explore whether PDLIM1 enhances the function of Tregs by stabilizing NRP1 or impairs the activity of CD8+ T cells, and how this balance affects survival outcomes in ccRCC. Overall, our findings support an angiogenesis-focused radiogenomic framework that complements conventional clinicopathological stratification within the evaluated cohorts. At the biological level, PDLIM1 emerged as a reproducible prognostic marker, with consistent associations across datasets and increased protein expression in ccRCC tissues and cell lines. Functionally, conditioned-medium experiments further indicate that tumor cell PDLIM1 can modulate endothelial tube formation, consistent with an effect on tumor cell-driven angiogenic signaling in vitro. Although these observations provide a biologically plausible link between PDLIM1 and angiogenesis, the underlying mechanisms remain to be defined. Future work should delineate PDLIM1-dependent secreted mediators (e.g., secretome proteomics/targeted ELISA), validate causality using rescue experiments, and confirm angiogenic effects in vivo using xenograft/orthotopic models with vascular readouts such as microvessel density and perfusion.

In the near term, this model could serve as a decision-support tool for postoperative risk stratification. Patients classified as higher risk by the combined model could be considered for closer surveillance intensity and more frequent imaging follow-up, whereas lower-risk patients might avoid unnecessary imaging burden, pending prospective validation. Importantly, the current study was not designed to guide treatment selection, because therapy response endpoints and decision impact analyses were not available. Nevertheless, an angiogenesis-focused radiogenomic phenotype may help prioritize patients for future studies evaluating response to anti-angiogenic or immunotherapy-containing regimens, and prospective trials will be required to determine whether model-informed management improves outcomes.

This study has limitations. First, the retrospective design may introduce bias, and prospective multicenter validation is needed. Second, while the CM-based tube formation assay supports a paracrine anti-angiogenic role of PDLIM1, this in vitro assay does not fully recapitulate in vivo angiogenesis. Future studies should identify the specific soluble mediators altered by PDLIM1 and perform rescue experiments. In vivo validation using xenograft/orthotopic models and micro-vessel density assessment would further strengthen causal inference. Third, although associations between PDLIM1 and immune features were observed, mechanistic studies are required to establish direct immunoregulatory roles.

## 5. Conclusions

In summary, we propose an angiogenesis-focused radiogenomic framework that integrates multi-cohort transcriptomics, scRNA-seq-informed angiogenesis programs, and preoperative CT radiomics to enable noninvasive and biologically interpretable prognostic stratification in ccRCC. This approach provides clinically relevant risk information that may complement conventional staging to support risk-adapted postoperative surveillance planning within the evaluated cohorts. Biologically, our integrative analyses nominate PDLIM1 as a prognostically favorable, angiogenesis-associated biomarker, supported by protein-level validation and in vitro evidence that tumor-cell PDLIM1 modulates endothelial tube formation through conditioned media. Future work should prioritize prospective multicenter validation, imaging protocol harmonization, and mechanistic studies (including secretome profiling and in vivo angiogenesis assays) to clarify causality and evaluate whether model-informed management improves clinical outcomes.

## Figures and Tables

**Table 1:** Univariate and multivariate analyses of clinical baseline characteristics.

Characteristic	Number		Univariable Cox Regression Analysis				Multivariable Cox Regression Analysis		
		Coefficient	HR	95% CI	*p* Value	Coefficient	HR	95% CI	*p* Value
**Age**									
≤60	73	reference	/	/	/	/	/	/	
>60	66	1.022	2.778	1.475–5.230	<0.01	0.869	2.384	1.239–4.587	<0.01
**Gender**									
Female	50	reference	/	/	/	/	/	/	/
Male	89	−0.468	0.627	0.347–1.131	0.121	/	/	/	/
**Stage**									
Localized	88	reference	/	/	/	reference	/	/	/
Locally advanced	30	1.178	3.247	1.562–6.750	<0.01	1.174	3.233	1.521–6.870	<0.01
Metastatic	21	1.892	6.632	3.263–13.480	<0.001	1.574	4.824	2.314–10.053	<0.001
**Grade**									
Low grade	60	reference	/	/	/	/	/	/	/
High grade	79	0.565	1.759	0.918–3.371	0.089	/	/	/	/
**Tumor laterality**									
Left	66	reference	/	/	/	reference	/	/	/
Right	73	−0.604	0.547	0.300–0.997	<0.05	−0.687	0.503	0.260–0.974	<0.05
**Race**									
Asian or black	12	reference	/	/	/	/	/	/	/
White	127	0.417	1.518	0.469–4.907	0.486	/	/	/	/

CI. confidence interval. HR. hazard ratio.

**Table 2:** The predictive performance of the prediction model based on the XGBoost algorithm in different datasets.

Model (Feature)	Model Performance (C-Index, 95%CI)								
	Training set (1-year)	Training set (3-year)	Training set (5-year)	Internal validation set (1-year)	Internal validation set (3-year)	Internal validation set (5-year)	External validation set (1-year)	External validation set (3-year)	External validation set (5-year)
Clinical model (Stage)	0.703 (0.581–0.824)	0.685 (0.580–0.790)	0.646 (0.526–0.766)	0.641 (0.540–0.742)	0.566 (0.360–0.773)	0.539 (0.335–0.742)	/	/	/
Radiogenomics mode (Radiogenomics score)	0.844 (0.703–0.985)	0.803 (0.683–0.924)	0.777 (0.658–0.896)	0.742 (0.640–0.844)	0.703 (0.535–0.871)	0.693 (0.501–0.885)	/	/	/
Combined model (Stage, Radiogenomics score)	0.880 (0.725–0.931)	0.816 (0.700–0.931)	0.789 (0.667–0.911)	0.864 (0.727–0.950)	0.758 (0.565–0.950)	0.736 (0.520–0.953)	0.800 (0.583–0.912)	0.726 (0.525–0.938)	0.703 (0.523–0.886)

CI: confidence interval.

## Data Availability

The raw data supporting the conclusions of this article will be made available by the authors on request.

## Supplementary Materials

The following supporting information can be downloaded at: https://www.mdpi.com/article/10.3390/cancers18050768/s1, Figure S1: The patient recruitment process for this study; Figure S2: Procedure for acquiring transcriptome data from the GEO microarray data; Figure S3: Procedure for acquiring transcriptome data from TCGA; Figure S4: Distribution of single-cell data and quality control process; Figure S5: The diagram of cell clustering and gene distribution; Figure S6: The enrichment analysis of angiogenesis-deg; Figure S7: Survival analysis of angiogenesis-hub genes; Figure S8: Correlation analysis between immune cells and risk scores; Figure S9: Correlation analysis between angiogenesis-hub genes and immune Infiltration status; Figure S10: Correlation analysis of risk score and somatic mutations; Figure S11: The distribution map of radiomics features with ICC > 0.75; Figure S12: The correlation coefficient plot of radiomics features associated with risk score; Figure S13: Kaplan—Meier curve analysis plot of the features used for constructing the predictive model; Figure S14: Calibration curves and decision curves of the models; Figure S15: Expression levels of PDLIM1 mRNA in tissues and its correlation analysis with clinical baseline characteristics; Figure S16: Gene set enrichment analysis of PDLIM1 in ccRCC; Figure S17: PDLIM1 mRNA expression across renal cancer cell lines in the CCLE database; Figure S18: Raw Western blot data of PDLIM1 expression in tissues (4 paired samples); Figure S19: Raw Western blot data of PDLIM1 expression in different cell lines; Figure S20: Raw Western blot data of PDLIM1 over-expression in 786-O cell line; Figure S21: Raw Western blot data of PDLIM1 knowdown in A-498 cell line; Table S1: The performance of the model based on different algorithms; Table S2: The Brier scores of prediction models developed using different algorithms; Table S3: Clinical baseline characteristics of the external validation cohort.

## Abbreviations

The following abbreviations are used in this manuscript:

ccRCC	Clear cell renal cell carcinoma
CI	Confidence interval
CM	Conditioned medium
Coxph	Cox regression
DCA	Decision Curve Analysis
DEGs	Differentially expressed genes
Enet	Elastic net
Gbm	Gradient boosting machine
GEO	Gene Expression Omnibus
GO	Gene Ontology
KEGG	Kyoto Encyclopedia of Genes and Genomes
KIRC	Kidney Renal Clear Cell Carcinoma
LASSO	Least absolute shrinkage and selection operator regression
Rsf	Random forest
Ridge	Ridge regression
ROIs	Regions of interest
RNA-seq	RNA sequencing
Stack	Stacked machine learning
TCGA	The Cancer Genome Atlas
TCIA	The Cancer Imaging Archive
OS	Overall survival
scRNA-seq	Single-cell RNA sequencing
vSMCs	Vascular Smooth Muscle Cells
Xgboost	extreme gradient boosting
