# Supplementary material for: Angiogenesis-Informed Preoperative CT Radiogenomics Predicts Overall Survival in Clear Cell Renal Cell Carcinoma: Development and External Validation
Source: Cancers (Basel). 2026 Feb 27;18(5):768. doi: 10.3390/cancers18050768 (PMC12985035; doi:10.3390/cancers18050768)
Supplement: Supplementary file 1 [file cancers-18-00768-s001.zip › Supplementary figures.pdf]

Supplementary Materials

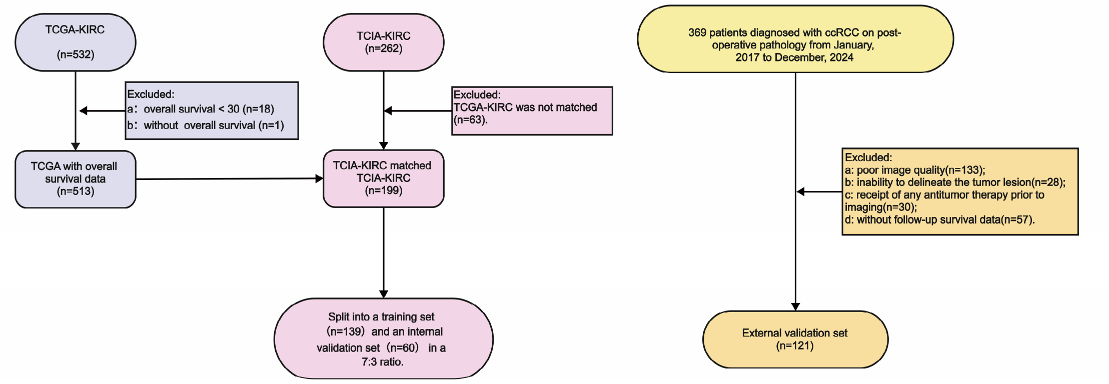

Figure S1. The patient recruitment process for this study.

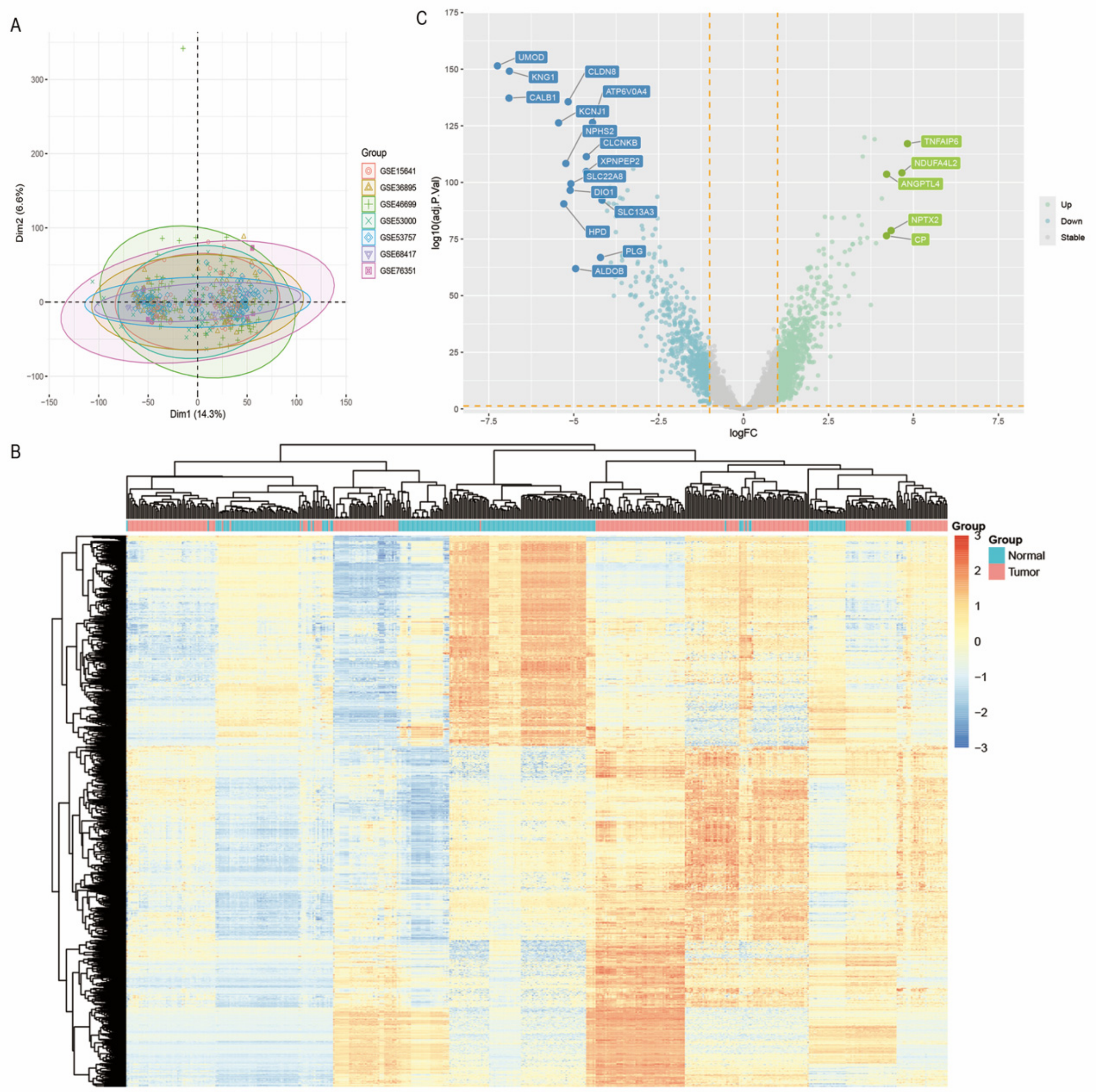

Figure S2. Procedure for acquiring transcriptome data from the GEO microarray data.

A. Visualization of the results of principal component analysis performed after batch effect removal from the data of seven microarrays. B. Heatmap depicting the grouping of transcriptomic genes derived from the microarray data. C. Volcano plot for differential analysis conducted based on the grouping of normal and tumor samples.

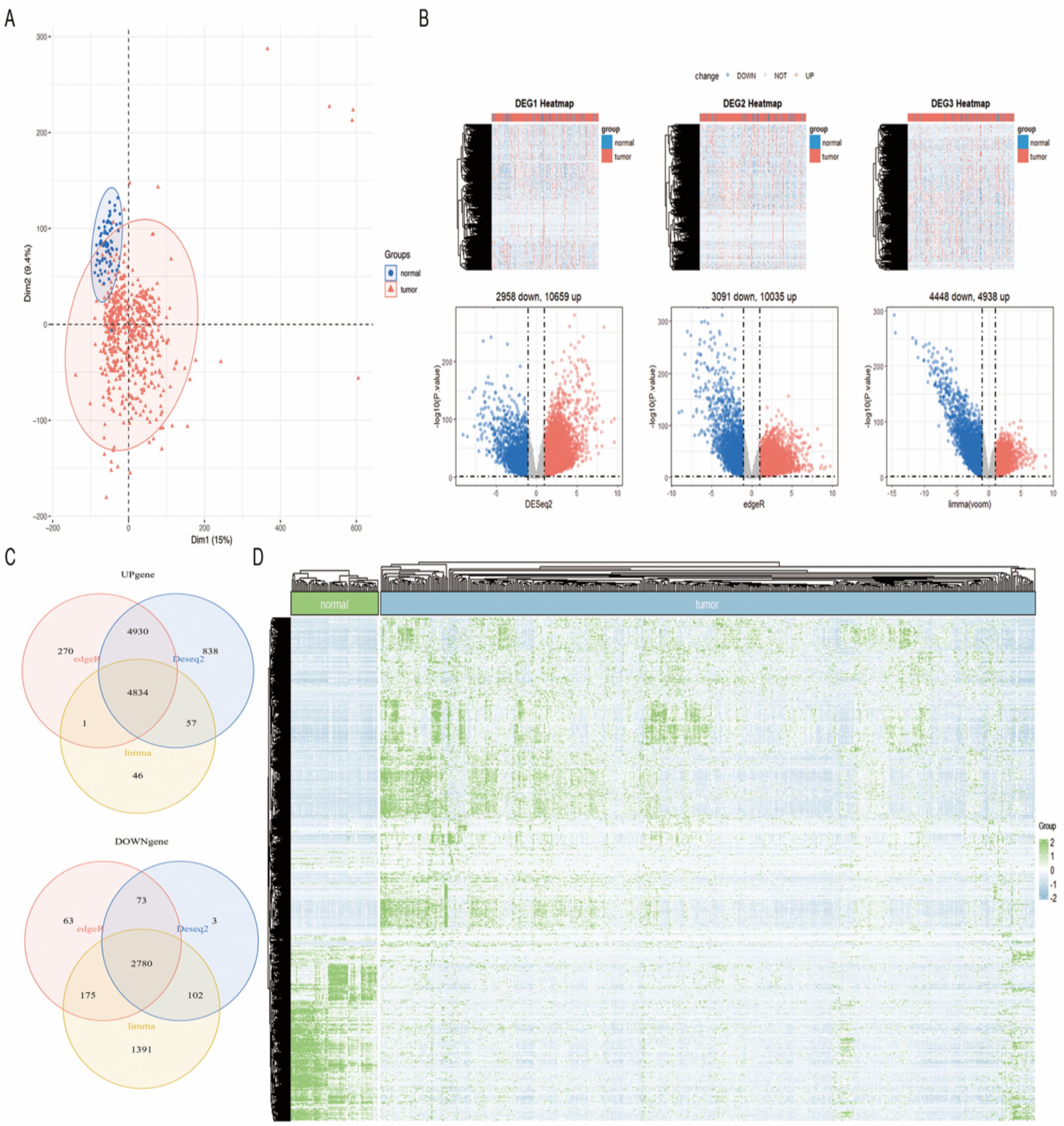

Figure S3. Procedure for acquiring transcriptome data from TCGA.

A. Principal component analysis plot of transcriptome data from the TCGA database; B. Grouping heat map and volcano plot of differentially expressed genes between normal and tumor groups analyzed by three algorithms; C. Venn diagram of the intersection of differential analysis based on the three algorithms; D. Grouping heat map of the differentially expressed genes after intersection.

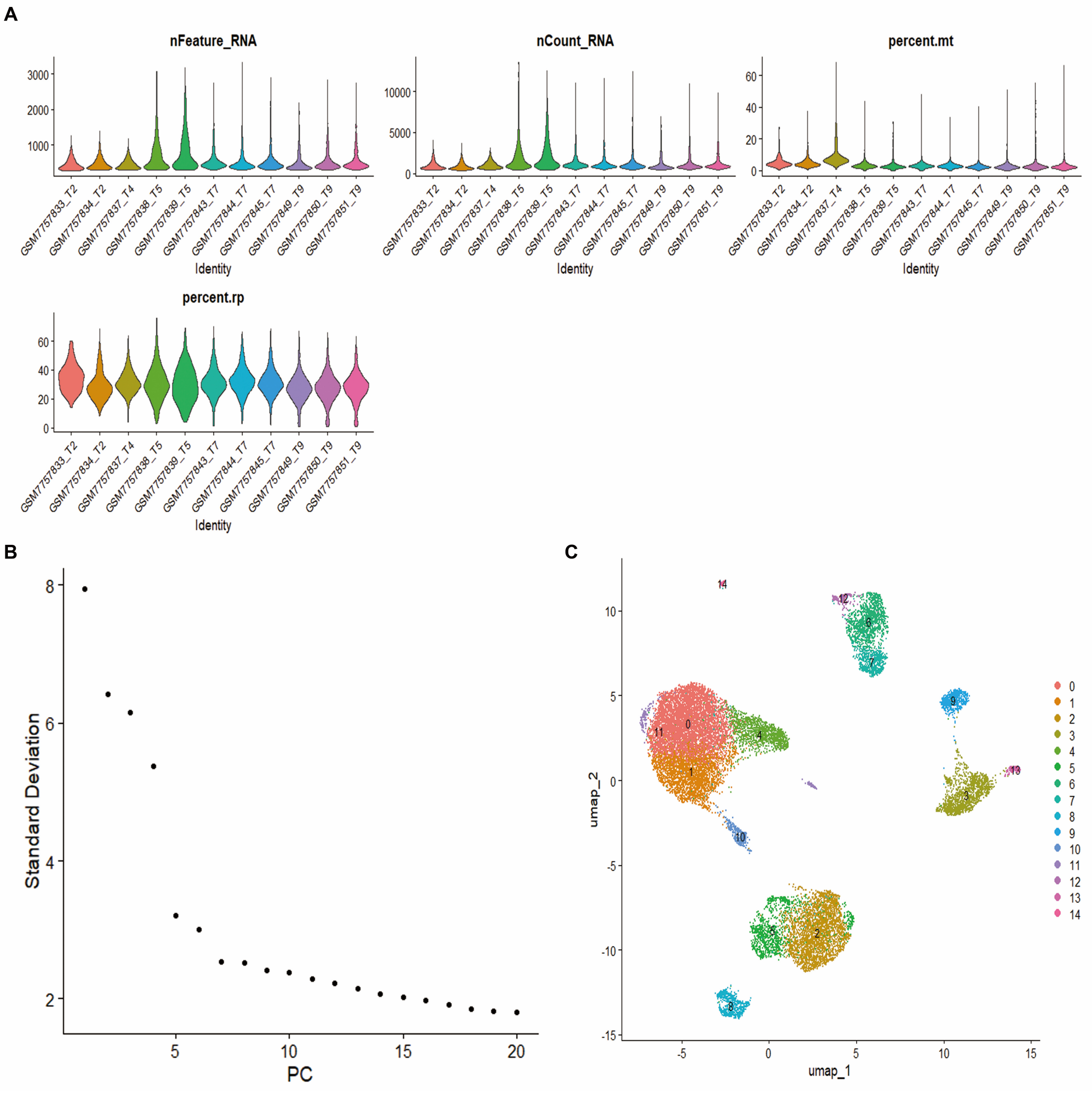

Figure S4. Distribution of single-cell data and quality control process.

A. nFeature_RNA represents the number of unique genes contained in each sample, nCount_RNA represents the total number of RNA molecules detected in each sample, percent.mt represents the percentage of mitochondrial genes in the total RNA of the sample, and percent.rp represents the percentage of ribosomal protein genes in the total RNA of the sample; B. Elbow graph; C. Unannotated cell clustering umap graph. PCA, principal component analysis. Umap, uniform manifold approximation and projection.

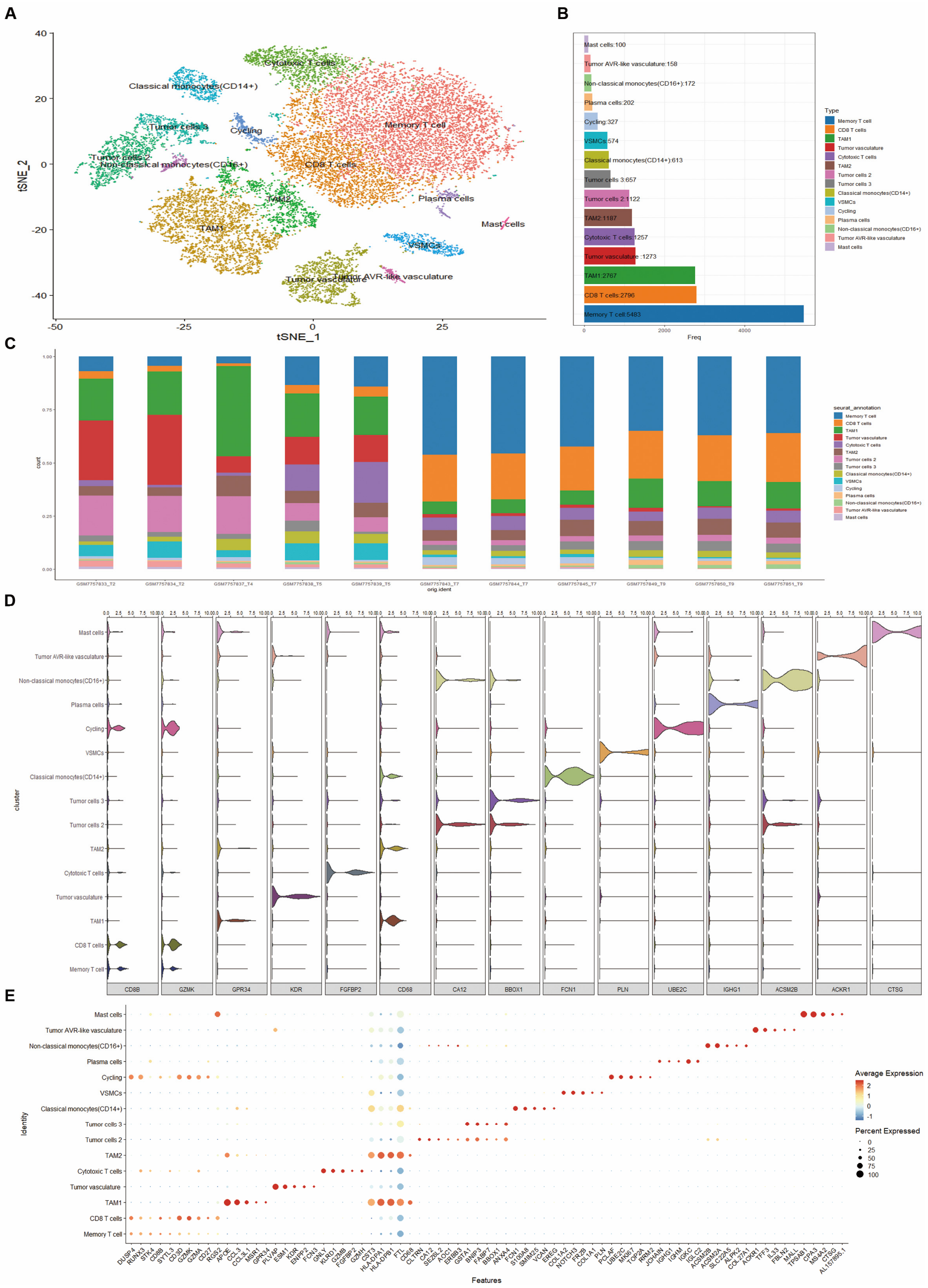

Figure S5. The diagram of cell clustering and gene distribution.

A. t-SNE plot illustrating cell clustering; B. Bar chart showing gene distribution across various cell types; C. Proportional distribution of cells across the samples; D. Violin plots representing marker genes for each cell cluster. TAM, tumor-associated macrophages; vSMCs, vascular smooth muscle cells.

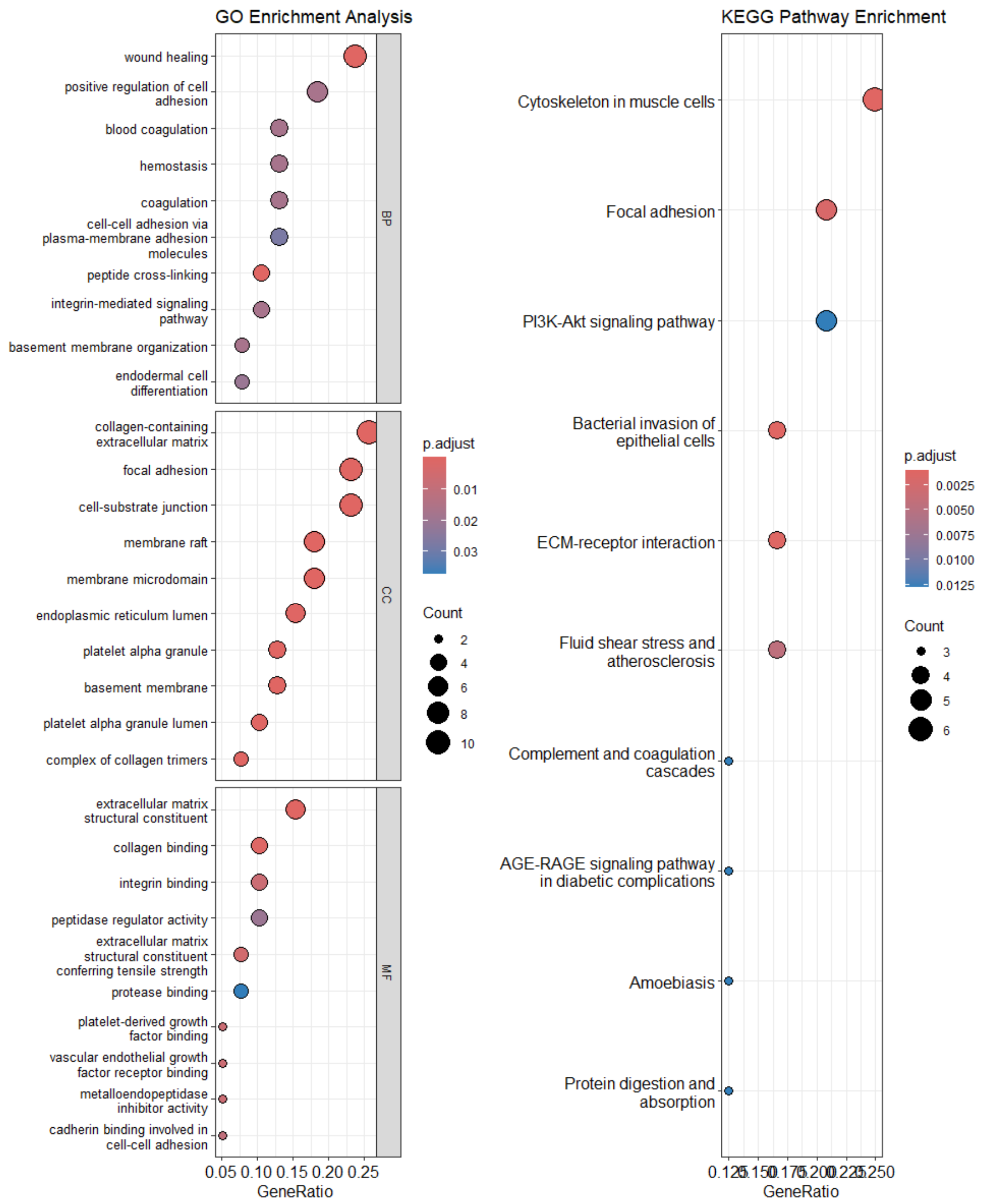

Figure S6. The enrichment analysis of angiogenesis-deg.

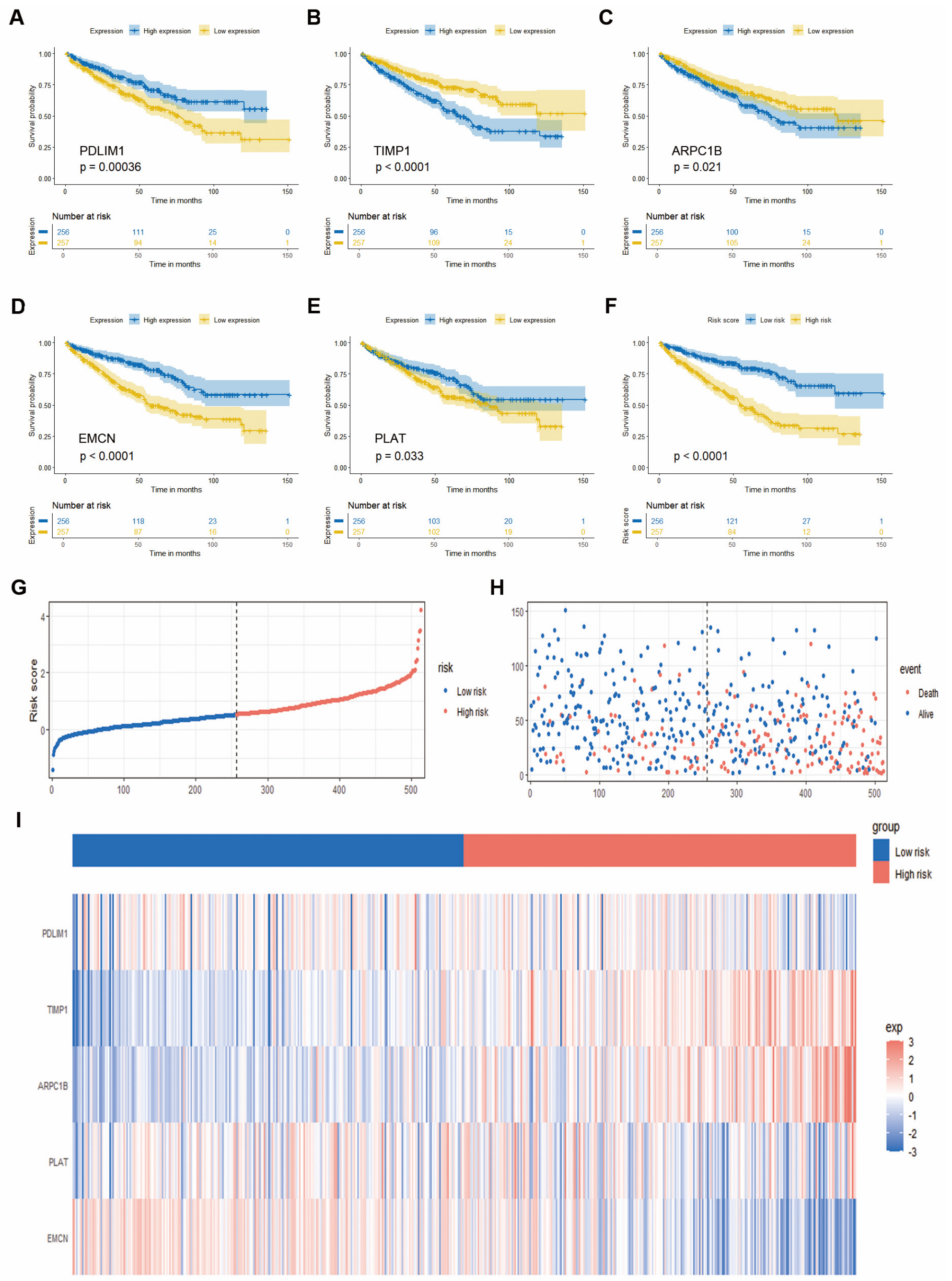

Figure S7. Survival analysis of angiogenesis-hub genes.

A-E. Kaplan-Meier curve analysis of angiogenesis-hub genes. F. Kaplan-Meier curve analysis of risk scores derived from angiogenesis-hub genes. G. Survival time of patients, sorted by risk score. H. Survival status of ccRCC patients, sorted by risk score. I. Heatmap showing the differences in the expression of seven angiogenesis-hub genes between high-risk and low-risk patients.

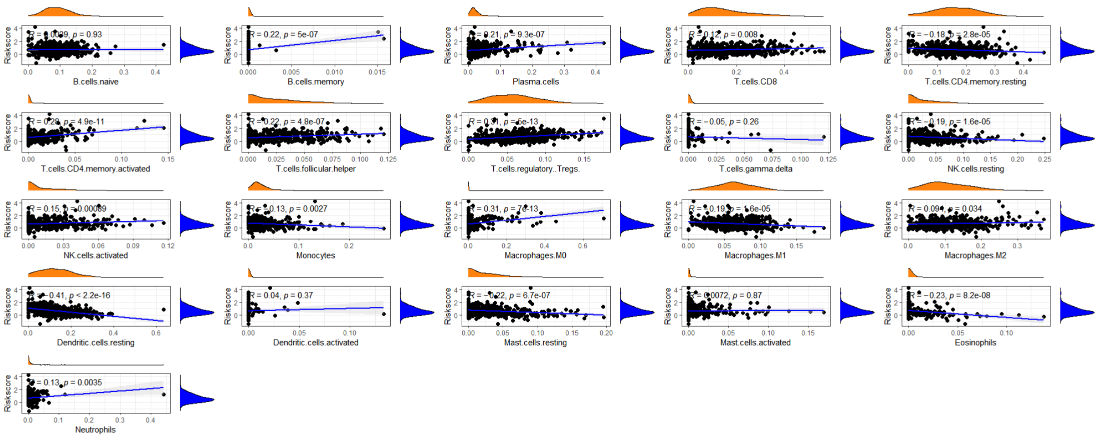

Figure S8. Correlation analysis between immune cells and risk scores.

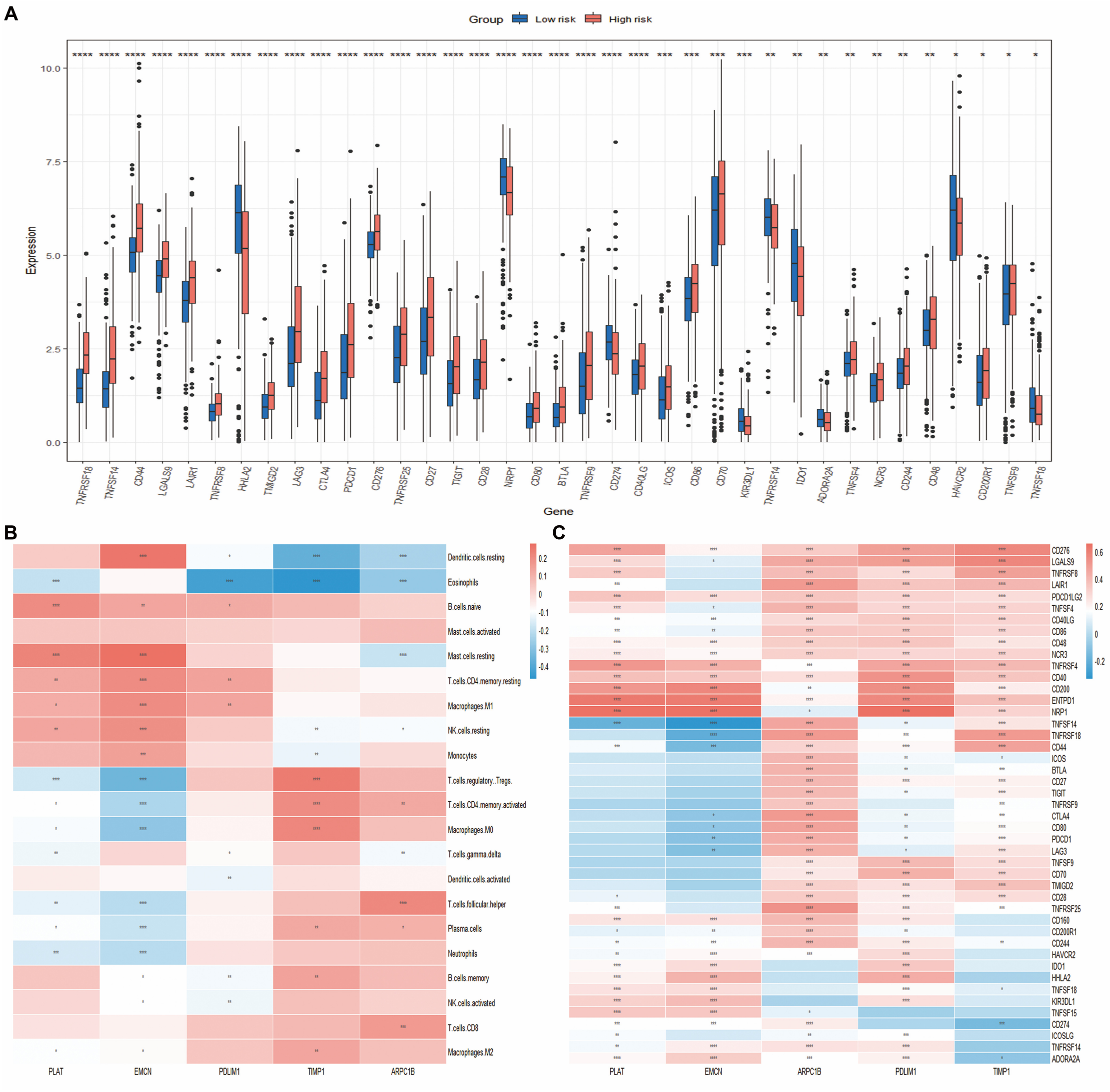

Figure S9. Correlation analysis between angiogenesis-hub genes and immune Infiltration status.

A. The expression of immune checkpoints between high-risk and low-risk groups; B. Correlation analysis plot of angiogenesis-hub genes and immune cells; C. Correlation analysis plot of angiogenesis-hub genes and immune checkpoints.

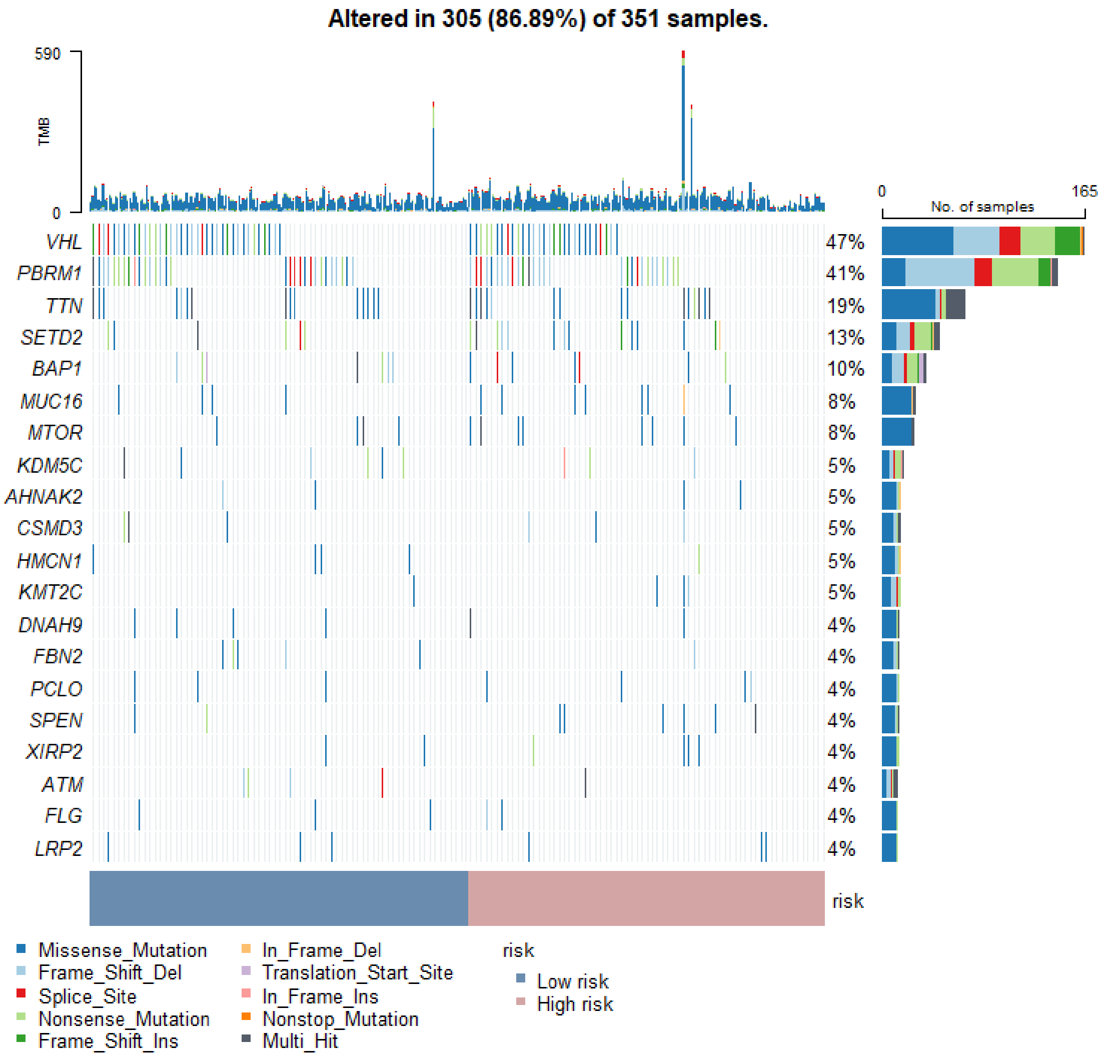

Figure S10. Correlation analysis of risk score and somatic mutations.

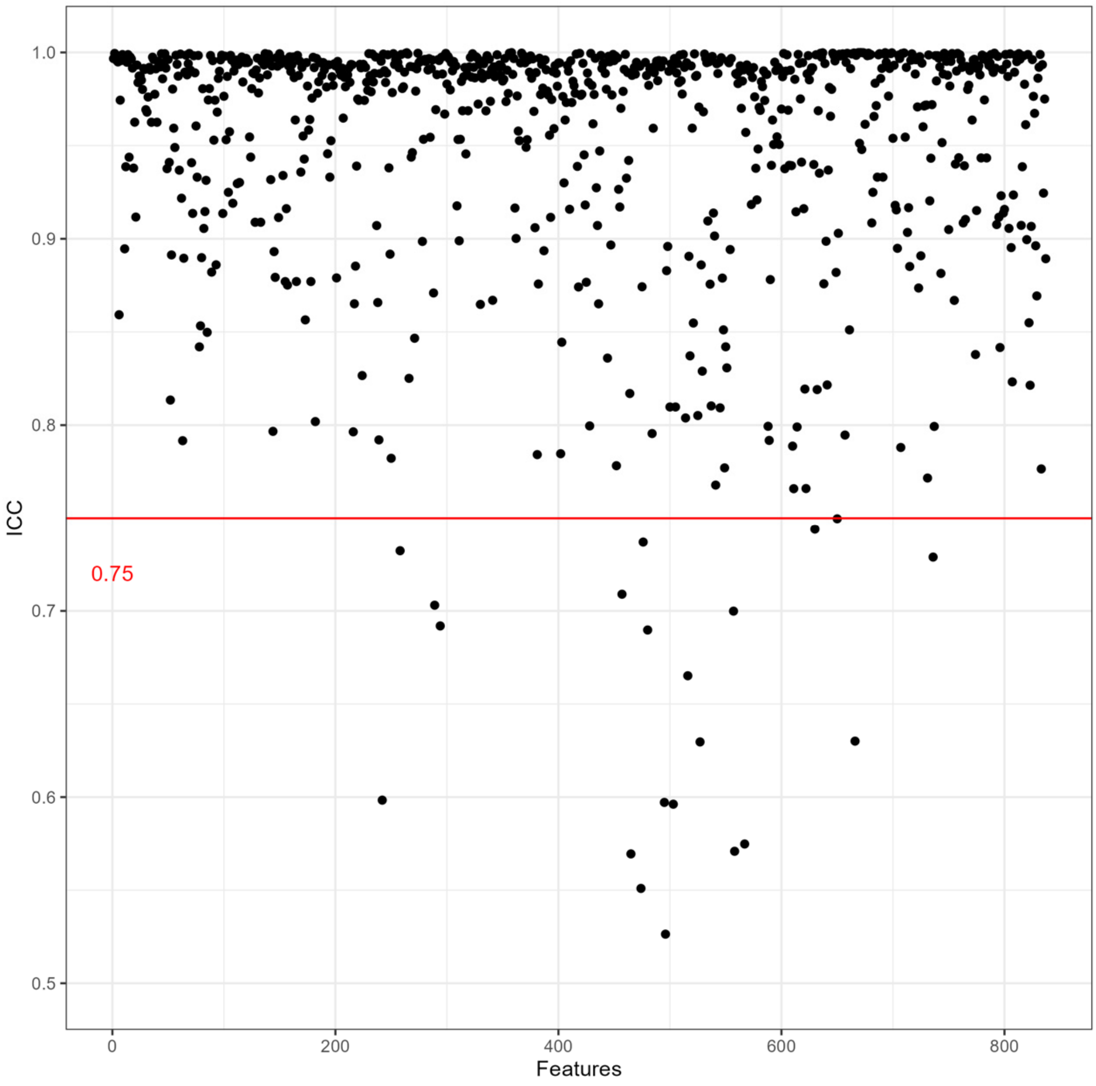

Figure S11. The distribution map of radiomics features with ICC > 0.75.

ICC, intraclass correlation coefficient.

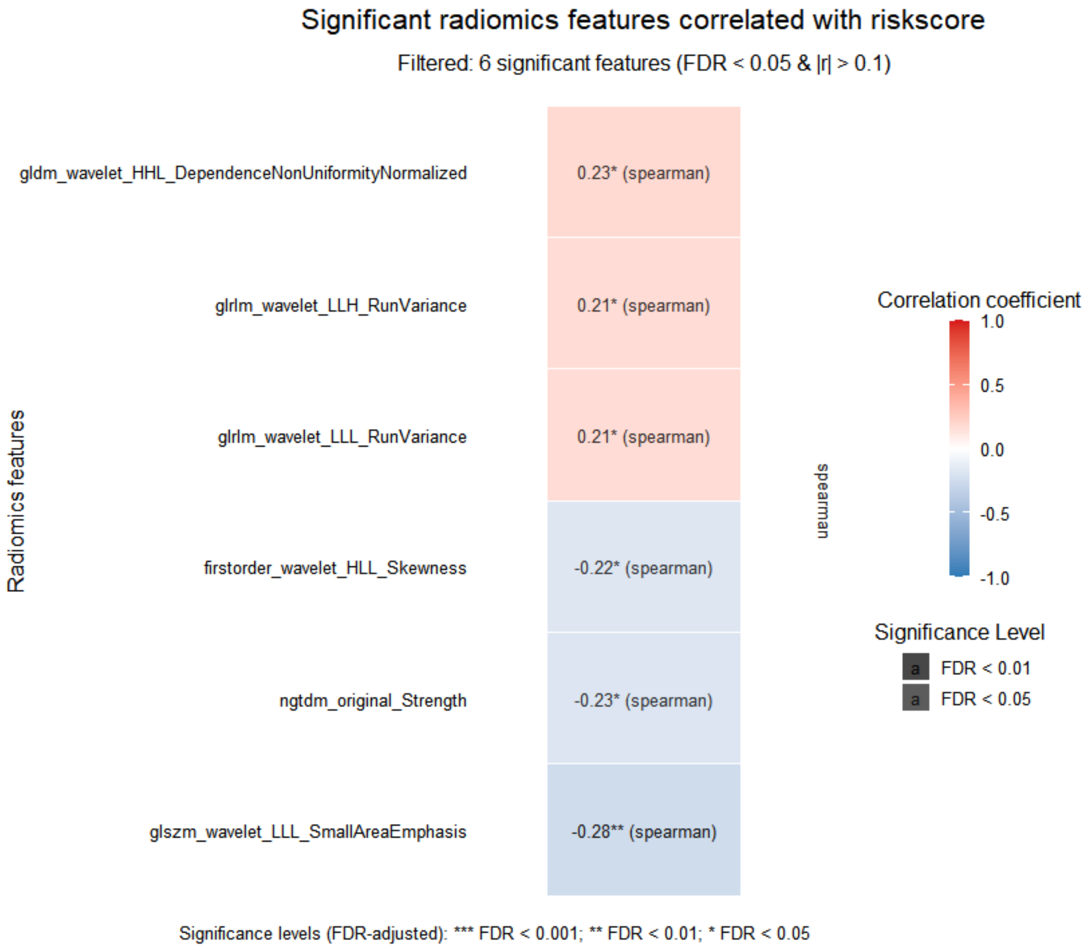

Figure S12. The correlation coefficient plot of radiomics features associated with risk score.

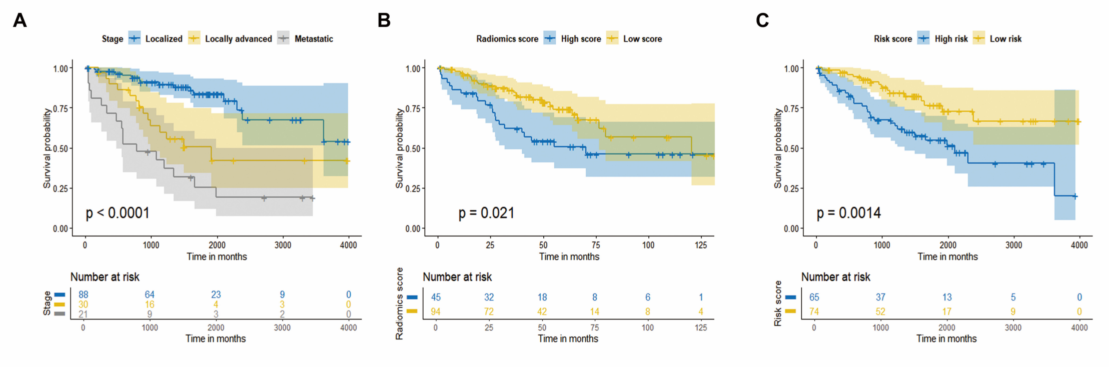

Figure S13. Kaplan - Meier curve analysis plot of the features used for constructing the predictive model.

A-C. Kaplan-Meier curve of stage, radiogenomics score, risk score.

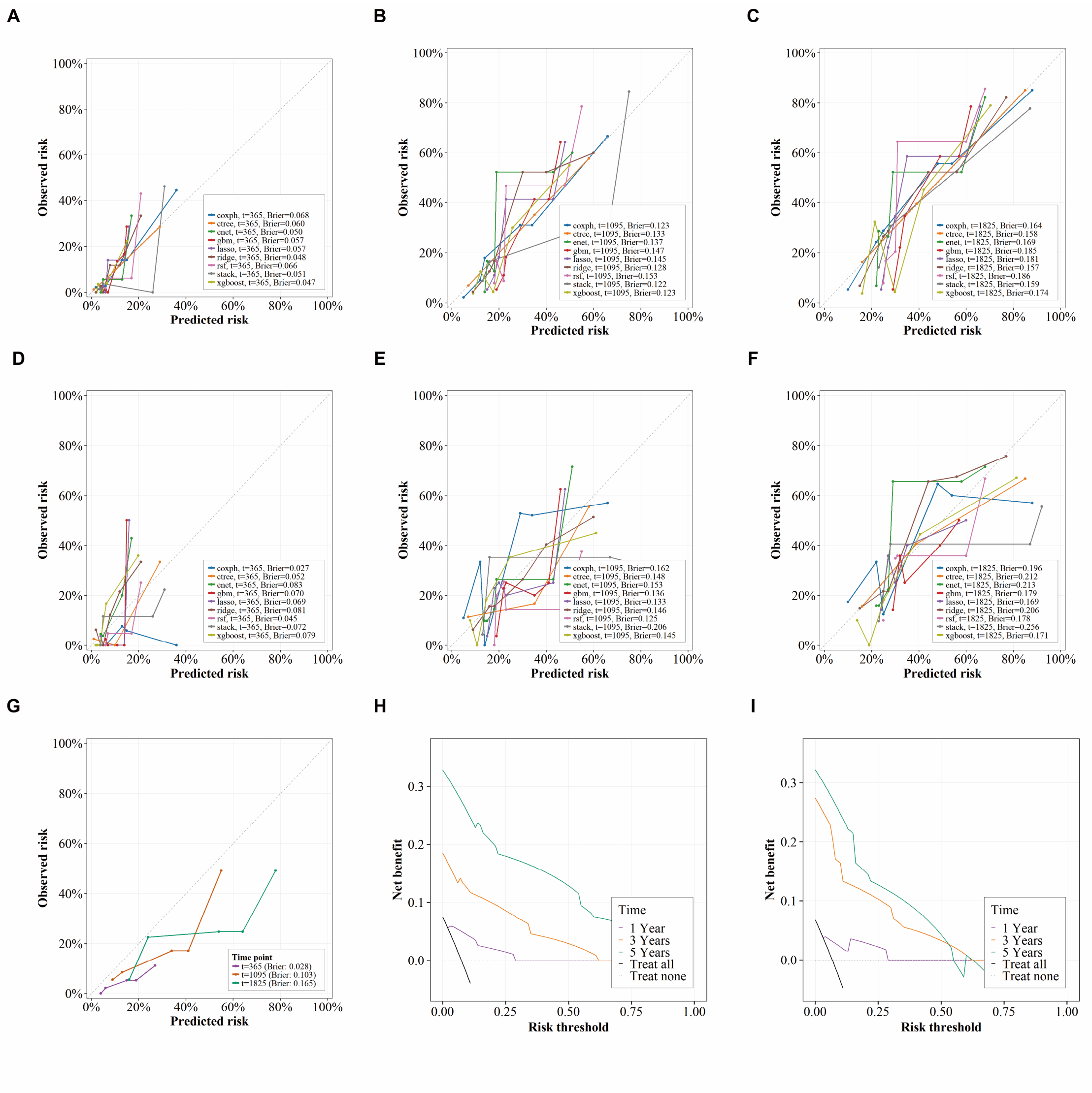

Figure S14. Calibration curves and decision curves of the models.

A-G. Calibration plots of the models developed in the training set (A-C), validated in the internal validation set (D-F), and in the external validation set (G). H-I. Decision curve analysis of training and internal validation sets.

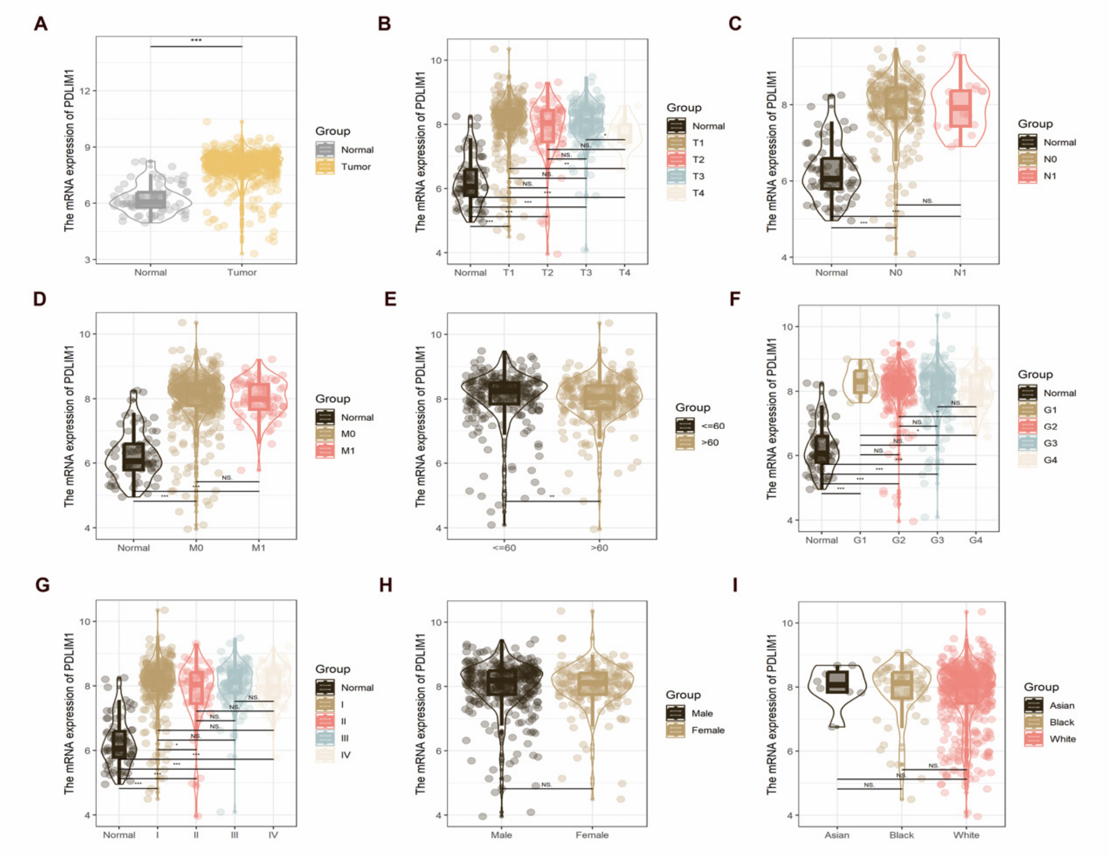

Figure S15. Expression levels of PDLIM1 mRNA in tissues and its correlation analysis with clinical baseline characteristics.

(A) mRNA expression levels of PDLIM1 in ccRCC tumors versus normal kidney tissues; B-I. Association of PDLIM1 expression with clinicopathological characteristics: (B) T stage, (C) N stage, (D) M stage, (E) Age groups, (F) Tumor grade, (G) AJCC stage, (H) Gender, (I) Race.

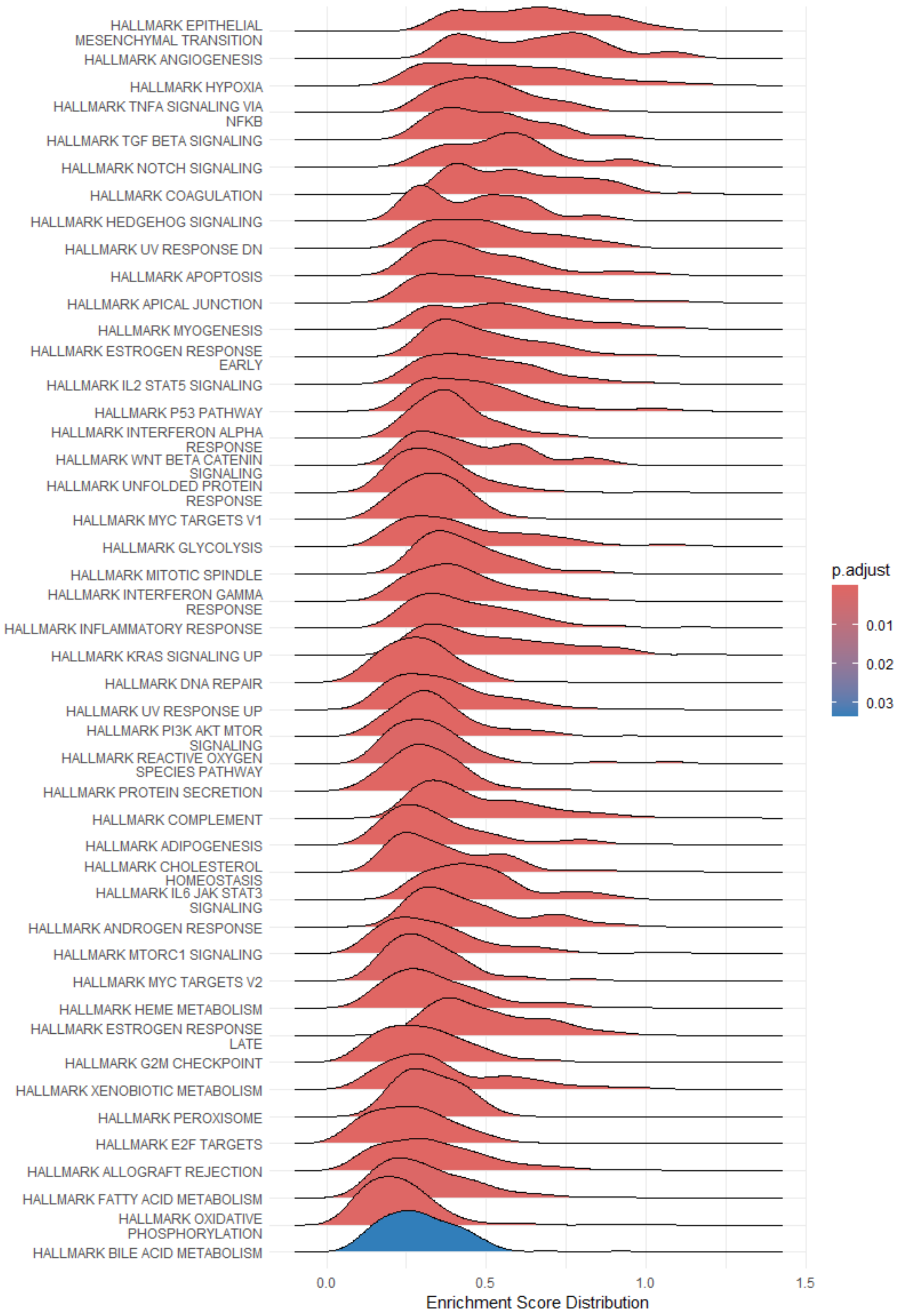

Figure S16. Gene set enrichment analysis of PDLIM1 in ccRCC.

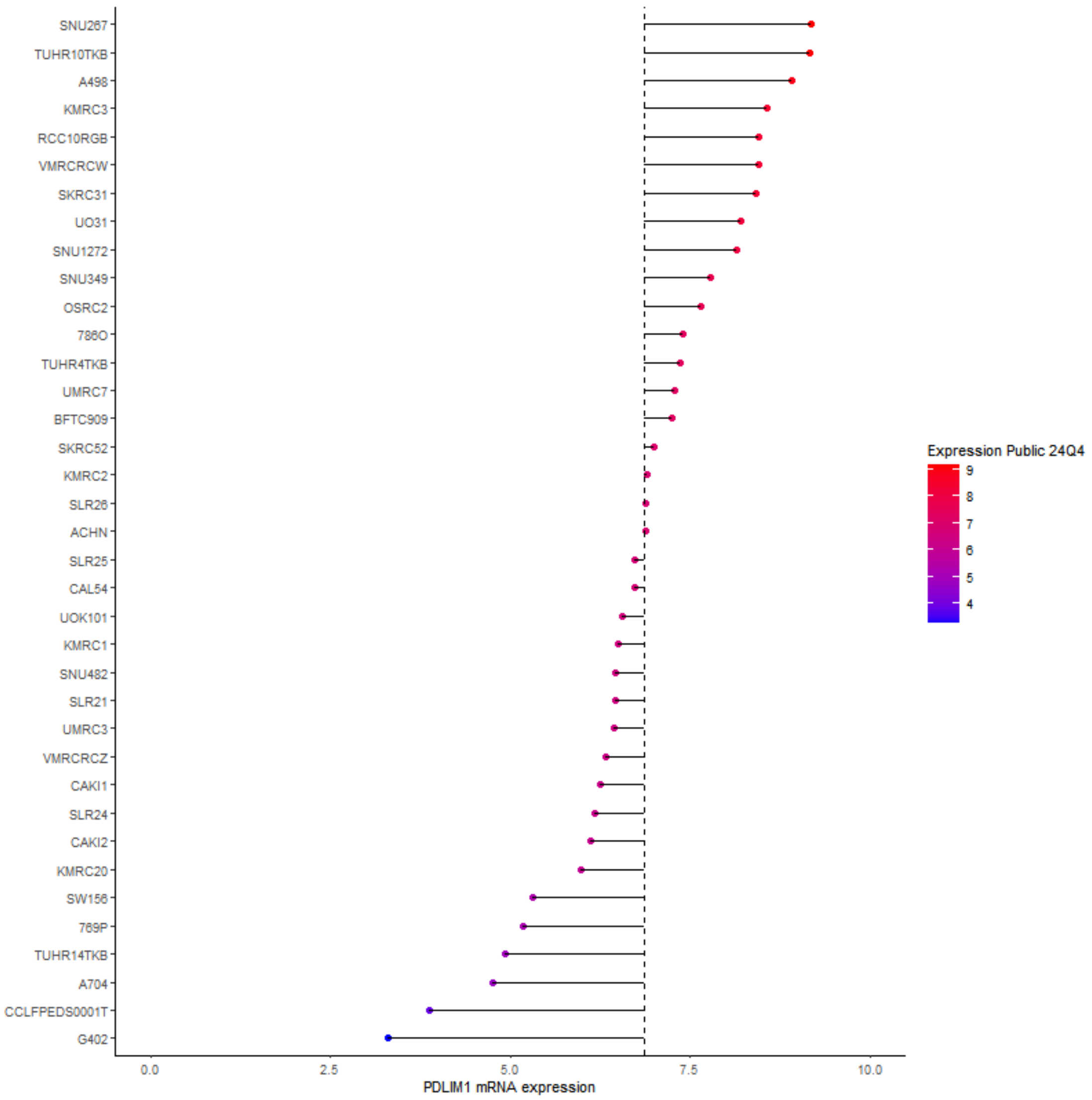

Figure S17. PDLIM1 mRNA expression across renal cancer cell lines in the CCLE database.

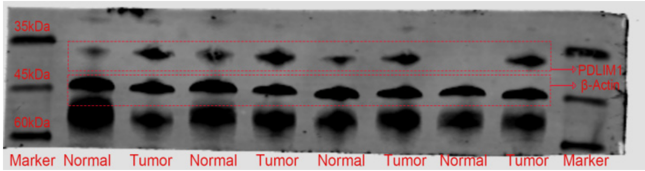

Figure S18. Raw Western blot data of PDLIM1 expression in tissues (4 paired samples)

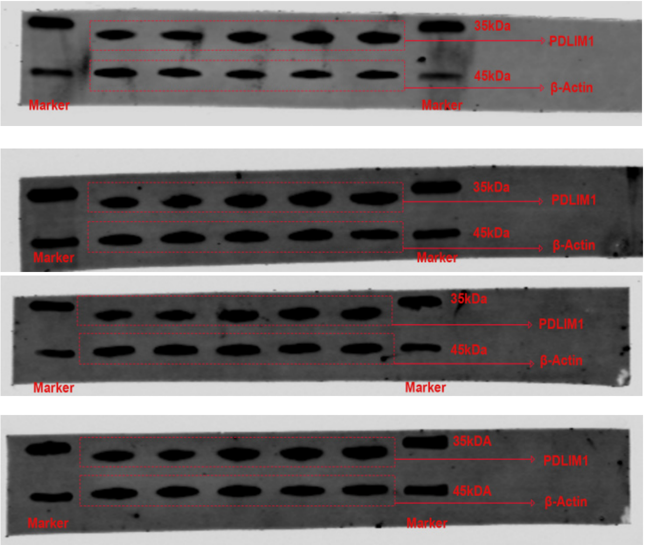

Figure S19. Raw Western blot data of PDLIM1 expression in different cell lines.

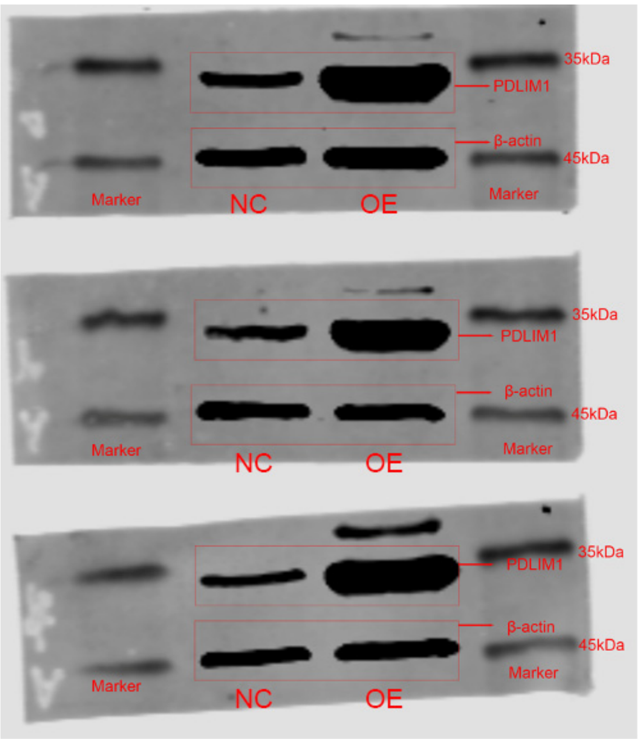

Figure S20. Raw Western blot data of PDLIM1 over-expression in 786-O cell line

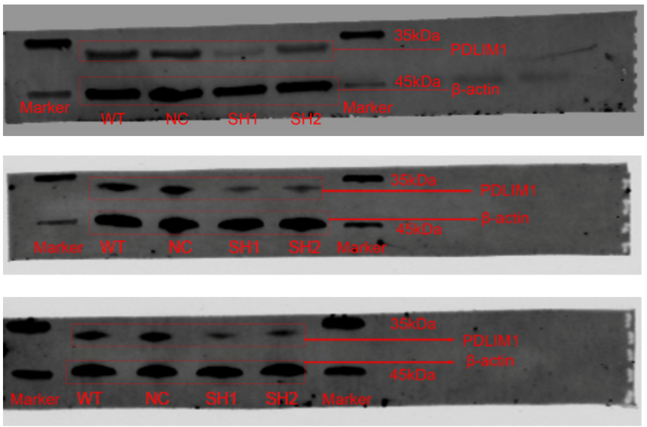

Figure S21. Raw Western blot data of PDLIM1 knowdown in A-498 cell line
