# Supplementary material for: Angiogenesis-Informed Preoperative CT Radiogenomics Predicts Overall Survival in Clear Cell Renal Cell Carcinoma: Development and External Validation
Source: Cancers (Basel). 2026 Feb 27;18(5):768. doi: 10.3390/cancers18050768 (PMC12985035; doi:10.3390/cancers18050768)
Supplement: Supplementary file 1 [file cancers-18-00768-s001.zip › Supplementary tables.pdf]

Table S1. The performance of the model based on different algorithms

Models	Model performance (C-index, 95%CI)					
	Training set (1-year)	Training set (3-year)	Training set (5-year)	Internal validation set (1-year)	Internal validation set (3-year)	Internal validation set (5-year)
Cox	0.848(0.704-0.992)	0.823(0.722-0.923)	0.799(0.683-0.914)	0.804(0.692-0.915)	0.709(0.492-0.926)	0.683(0.470-0.897)
Tree	0.823(0.677-0.970)	0.787(0.671-0.902)	0.786(0.677-0.905)	0.795(0.475-0.915)	0.694(0.480-0.909)	0.690(0.488-0.891)
Lasso	0.848(0.729-0.968)	0.796(0.684-0.907)	0.795(0.684-0.905)	0.818(0.508-0.895)	0.791(0.614-0.968)	0.782(0.588-0.977)
Xgboost	0.880(0.725-0.931)	0.816(0.700-0.931)	0.789(0.667-0.911)	0.864(0.727-0.950)	0.758(0.565-0.950)	0.736(0.520-0.953)

Gbm	0.848(0.725-0.972)	0.796(0.680-0.911)	0.795(0.681-0.909)	0.818(0.508-0.900)	0.791(0.614-0.968)	0.782(0.588-0.977)
Ridge	0.881(0.765-0.997)	0.811(0.697-0.925)	0.806(0.693-0.918)	0.779(0.493-0.900)	0.750(0.566-0.934)	0.717(0.505-0.903)
Enet	0.894(0.800-0.988)	0.803(0.685-0.921)	0.787(0.669-0.906)	0.779(0.493-0.800)	0.745(0.560-0.931)	0.693(0.477-0.909)
Rsf	0.851(0.693-1.000)	0.796(0.679-0.912)	0.787(0.667-0.907)	0.851(0.653-1.000)	0.775(0.611-0.939)	0.721(0.531-0.912)
Stack	0.828(0.640-0.900)	0.766(0.634-0.898)	0.760(0.637-0.882)	0.802(0.643-0.962)	0.696(0.532-0.859)	0.665(0.473-0.857)

Coxph. Cox regression. Ctree. Decision tree. Lasso. Least absolute shrinkage and selection operator regression. Xgboost. extreme gradient boosting. Gbm. Gradient boosting machine. Ridge, Ridge regression. Enet. Elastic net. Rsf. Random forest. Stack. Stacked machine learning. CI. Confidence interval.

Table S2. The Brier scores of prediction models developed using different algorithms

Models	Brier score						
	Training set (1-year)	Training set (3-year)	Training set (5-year)	Internal validation set (1-year)	Internal validation set (3-year)	Internal validation set (5-year)	Average score
Cox	0.068	0.123	0.164	0.0268	0.162	0.196	0.123
Tree	0.06	0.133	0.158	0.0523	0.148	0.212	0.127
Lasso	0.057	0.145	0.181	0.0686	0.133	0.169	0.126
Xgboost	0.047	0.123	0.174	0.0792	0.145	0.171	0.123
Gbm	0.057	0.147	0.185	0.0704	0.136	0.179	0.129
Ridge	0.048	0.128	0.157	0.0810	0.146	0.206	0.128
Enet	0.05	0.137	0.169	0.0831	0.153	0.213	0.134
Rsf	0.066	0.153	0.186	0.0331	0.139	0.188	0.126

Stack	0.051	0.122	0.159	0.0718	0.206	0.256	0.144

Coxph. Cox regression. Ctree. Decision tree. Lasso. Least absolute shrinkage and selection operator regression. Xgboost. extreme gradient boosting. Gbm. Gradient boosting machine. Ridge, Ridge regression. Enet. Elastic net. Rsf. Random forest. Stack. Stacked machine learning. CI. Confidence interval.

Table S3. Clinical baseline characteristics of the external validation cohort

**Features**		N（%）
**Stage**	Localized	93 (76.9%)
	Locally advanced	17 (14.0%)
	Metastatic	11 (9.1%)
**Radiogenomics score**	High score	72 (59.5%)
	Low score	49 (40.5%)

**Overall survival**	Median follow-up, months	38.7
	Deaths (events), n (%)	16 (13.2)

N. number of patients.
